# Nonprecious Triple-Atom Catalysts with Ultrahigh Activity for Electrochemical Reduction of Nitrate to Ammonia: A DFT Screening

**DOI:** 10.1021/acsami.4c17726

**Published:** 2025-01-10

**Authors:** Xiangyi Zhou, Mohsen Tamtaji, Weijun Zhou, William A. Goddard, GuanHua Chen

**Affiliations:** † Department of Chemistry, The University of Hong Kong, Pokfulam Road, Hong Kong SAR 999077, China; ‡ Hong Kong Quantum AI Lab Limited, Pak Shek Kok, Hong Kong SAR 999077, China; § QuantumFabless Limited, Pak Shek Kok, Hong Kong SAR 999077, China; ∥ Materials and Process Simulation Center, MC 139-74, California Institute of Technology, Pasadena, California 91125, United States

**Keywords:** ammonia synthesis, non-noble metal-based catalysts, In silico catalyst design, beyond single-atom catalysts, breaking scaling relationship

## Abstract

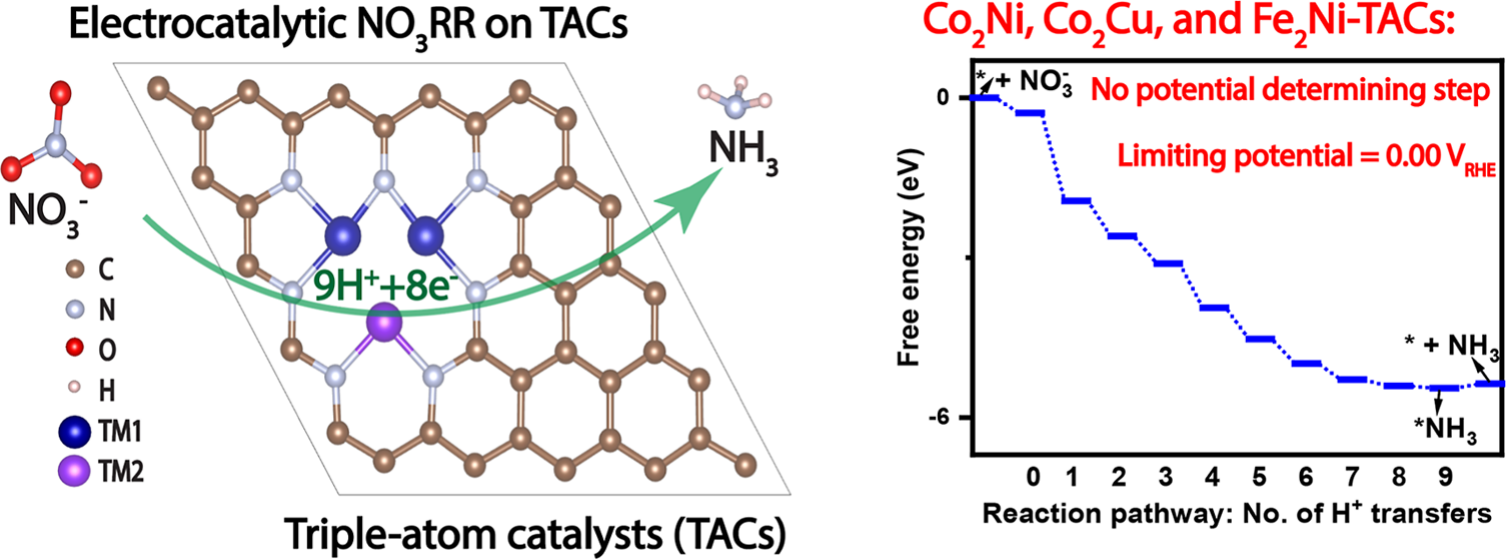

Electrochemical nitrate reduction to ammonia (NO_3_RR) is promising to not only tackle environmental issues caused by nitrate but also produce ammonia at room temperatures. However, two critical challenges are the lack of effective electrocatalysts and the understanding of related reaction mechanisms. To overcome these challenges, we employed first-principles calculations to thoroughly study the performance and mechanisms of triple-atom catalysts (TACs) composed of transition metals (including 27 homonuclear TACs and 4 non-noble bimetallic TACs) anchored on N-doped carbon (NC). We found five promising candidates possessing not only thermodynamic and electrochemical stability, but also high activity and selectivity for ammonia production. Among them, non-noble homonuclear Ni_3_@NC TAC show high activity with low theoretical limiting potential of −0.31 *V*_RHE_. Surprisingly, bimetallic Co_2_Ni@NC, Co_2_Cu@NC, and Fe_2_Ni@NC TACs show ultrahigh activity with theoretical limiting potentials of 0.00 *V*_RHE_, without a potential determining step in the whole reaction pathways, representing the best theoretical activity been reported up to date. These promising candidates are facilitated by circumventing the limit of scaling relationships, a well-known obstacle for single-atom catalysts. This study indicates that designing suitable TACs can be a promising strategy for efficiently electro-catalyzing NO_3_RR and breaking the limit of the scaling relationship.

## 1 Introduction

Nitrate pollution arising from industrialization and agricultural activities poses significant risks to aquatic ecosystems and human health, contributing to diseases like cancer and blue baby syndrome.^1−3^ Consequently, effectively removing surplus nitrate ions from contaminated water is imperative for sustainable development. Recent findings^4−10^ suggest that electrocatalytic reduction of nitrate to ammonia (NO_3_RR) serves as a means to kill two birds with one stone.

Ammonia (NH_3_) is essential to nitrogen-based fertilizers and shows significant potential as a hydrogen-rich fuel for industry.^3^ Currently, NH_3_ is predominantly produced at a large scale using the energy-intensive Haber–Bosch (HB) process, which operates under high temperature conditions (400–600 °C) and pressures (150–350 atm).^11,12^ Moreover, the hydrogen required for the HB process usually originates from steam methane reforming (CH_4_ + H_2_O → CO + 3H_2_), resulting in the release of environmentally harmful CO and CO_2_. This reliance on fossil fuels underscores the need for more sustainable ammonia production methods. In view of this, electrochemical synthesis of ammonia from nitrate reduction (NO_3_RR) under ambient conditions (NO_3_^–^ + 9H^+^ + 8e^–^ → NH_3_ + 3H_2_O) emerges as a promising approach. This is due to the ready availability of the nitrate anion in nature and its comparatively low N=O bond dissociation energy (2.1 eV).^13,14^ Besides, this strategy would simultaneously reduce nitrate pollutant. However, NO_3_RR is usually sluggish and complex with undesired byproducts, such as NO and NO_2_^6,9^ Therefore, understanding the atomic-level mechanisms governing NO_3_RR pathways is crucial for designing new highly efficient electrocatalysts.^15,16^ Consequently, designing active, selective, stable, and cost-effective electrocatalysts for NO_3_RR continues to pose a significant challenge.^17^

Increasing amounts of research has focused on designing atomically dispersed metal catalysts supported by 2D substrates, initially from single atom catalysts (SACs),^18−23^ then to dual-atom catalysts (DACs),^24,25^ and even triple-atom catalysts (TACs).^26−31^ Compared with traditional metal-based catalysts, atomically dispersed metal catalysts require lower cost due to much higher utilization of metal atoms. Some of them demonstrate outstanding catalytic activity across a wide range of electrochemical reactions, such as the oxygen reduction reaction (ORR),^23,32−36^ the oxygen evolution reaction OER,^33,36^ the N_2_ reduction reaction (N_2_RR),^37^ and the CO_2_ reduction reaction (CO_2_RR).^38−40^ For NO_3_RR, SACs generally show limited activity due to the confinement of scaling relationship among intermediates.^5,6^ However, a few DACs are promising for NO_3_RR catalysis with high activity and selectivity. For example, Cr_2_ on expanded phthalocyanine (Cr_2_-Pc) is predicted theoretically to be a superior catalyst with a limiting potential of −0.02 V.^8^ Theoretical prediction and experimental validation show that the Cu dual-atom site with three nitrogen coordination is excellent for NO_3_RR with Faradaic efficiency (FE) of 97.4%.^7^ However, the development of NO_3_RR electrocatalysts is still in its infancy. Notably, there have been no reported TACs for NO_3_RR to NH_3_ to date, although some TACs are promising catalysts for other reactions, such as Fe_3_-TAC on graphdiyne for N_2_RR,^29^ Fe_3_-TAC on N-doped graphene for rapid CO electroreduction to propylene,^41^ and Cu_3_-TAC on the S-terminated MoSTe surface for CO_2_ electroreduction to CH_4_.^42^ Therefore, we are inspired to screen M_3_-TACs for NO_3_RR and to investigate the corresponding atomic-level mechanisms.

This study represents the inaugural comprehensive evaluation of the stability, selectivity, and electrocatalytic activity of M_3_-TACs on N-doped carbon (NC) for NO_3_RR. Employing in silico screening, we proposed both homonuclear and bimetallic M_3_-TACs for enhanced NO_3_RR toward NH_3_. Our computational findings revealed that non-noble bimetallic Co_2_Ni@NC, Co_2_Cu@NC, Fe_2_Ni@NC, Co_2_Fe@NC, and homonuclear Ni_3_@NC TACs, exhibit considerable promise as NO_3_RR electrocatalysts, characterized by low limiting potential and high selectivity. We investigated the potential of TACs to break the linear scaling relationship between reaction intermediates. We analyzed the electronic influence of secondary metal atoms in bimetallic Co_2_M′@NC TACs.

## 2 Computational Methods

We conducted all DFT calculations using VASP 5.4.4 software, applying a plane-wave cutoff energy of 500 eV and employing the Perdew–Burke–Ernzerhof (PBE) exchange–correlation functional within the generalized gradient approximation (GGA) framework, with spin polarization.^43−46^ van der Waals interactions were accounted for using the Grimme D3 method.^47,48^ Additionally, we utilized VASPsol to simulate the implicit solvation effect of water.^49,50^ Convergence thresholds were set at 0.04 eV/Å for force and 10^–6^ eV for energy and electronic structure.

N-doped carbon was modeled by a graphene *p*(5 × 5) supercell with a lattice constant of 12.28 Å, and a vacuum space of 20 Å was implemented to prevent interactions between adjacent periodic images. During structural relaxation, three carbon atoms in each TAC model are fixed to reduce the distortion and corresponding energy error (as shown in the relaxed structure file in Note S2 of the Supporting Information). Brillouin zone sampling followed the Monkhorst–Pack scheme and utilized 3 × 3 × 1 and 5 × 5 × 1 *k*-point grids, for structural optimization and single-point energy calculations, respectively. To compute the Gibbs free energy change (Δ*G*) for each step during nitrate reduction, we employed the computational hydrogen electrode (CHE) model, as follows^51^
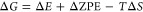
1Here, Δ*E* represents the electronic energy difference, ΔZPE stands for the zero-point energy, *T* denotes the temperature (298.15 K), and Δ*S* accounts for entropy corrections.

To circumvent the direct calculation of the energy of charged NO_3_^–^, we adopted gaseous HNO_3_ as a reference. The adsorption energy of NO_3_^–^ (ΔG_*NO_3__) was determined as follows
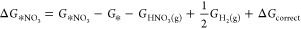
2In this equation, G_*NO_3__, G_*_, , and  denote the intrinsic Gibbs free energies of adsorbed NO_3_^–^, bare TAC, gas-phase HNO_3_, and gas-phase H_2_, respectively. Additionally, Δ*G*_correct_ represents the correction for NO_3_^–^ adsorption energy, set to 0.392 eV.

The total reaction of NO_3_RR can be expressed as NO_3_^–^ + 9H^+^ + 8e^–^ → NH_3_ + 3H_2_O. Note S1 shows the detailed formula of all elementary process observed during the search of minimum energy reaction pathways.

## 3 Results and Discussions

### 3.1 Structure and Stability of TACs

**Figure 1:**
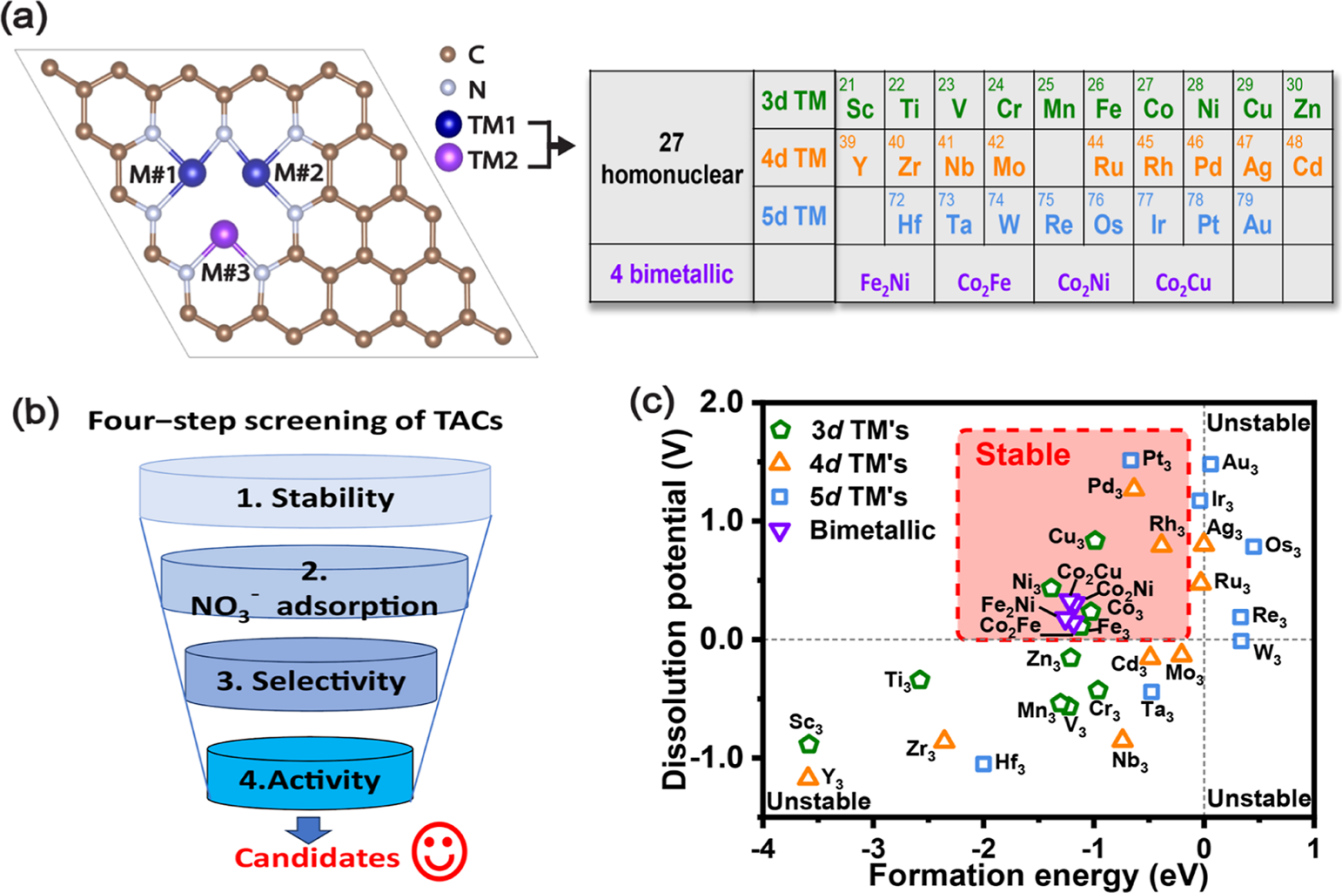
(a) Schematics of studied TACs. (b) Four steps for screening promising TACs for NO_3_RR. (c) Stabilities of metal trimers in studied TACs.

Figure 1a presents the configuration of studied M_3_-TACs. Such a TAC model is based on another theoretical research,^41^ and similar to some experimental results.^26,52^ Specifically, Fe_3_@NC^52^ and ZnCoFe@NC TAC with similar structures have been synthesized and their catalytic applications have been explored, suggesting the feasibility of experimentally synthesizing understudied TAC structures. Our work can bring fundamental insights into the future experimental synthesis of the proposed TACs. In this TAC model, metal trimers are located at the six carbon vacancies, and each metal trimer is anchored by six pyridine nitrogen atoms and one amino nitrogen atom in N-doped carbon, denoted as M_3_@NC TACs (M_3_-TACs for brevity). Each trimer consists of three metal atoms bonded together, designated as M#1 for the metal site in the upper left, M#2 for the metal site in the upper right, and M#3 for the metal site in the lower middle. We systematically considered the 27 homonuclear-metallic (Sc_3_, Ti_3_, V_3_, Cr_3_, Mn_3_, Fe_3_, Co_3_, Ni_3_, Cu_3_, Zn_3_, Y_3_, Zr_3_, Nb_3_, Mo_3_, Ru_3_, Rh_3_, Pd_3_, Ag_3_, Cd_3_, Hf_3_, Ta_3_, W_3_, Re_3_, Os_3_, Ir_3_, Pt_3_, and Au_3_) and 4 bimetallic trimers (Fe_2_Ni, Co_2_Fe, Co_2_Ni, and Co_2_Cu).

In the hope of finding promising TACs for electrocatalytic reduction of nitrate to ammonia, we adopted a four-step screening to theoretically investigate the stability, selectivity, and activity of these TACs, as shown in Figure 1b.

To assess the thermodynamic and electrochemical stability of these TACs, we computed their formation energy (*E*_form_) and dissolution potential (*U*_diss_) as detailed below^8,53^
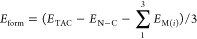
3
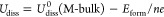
4In the equation, *E*_TAC_ stands for the total energy of the TAC, *E*_N-G_ denotes the total energy of the nitrogen-doped carbon, and *E*_M(*i*)_ represents the atomic energy of metal M(*i*) in its most stable bulk state. *U*_diss_^0^(M-bulk) represents the standard dissolution potential of the bulk metals, while *n* corresponds to the stoichiometric coefficient representing the number of electrons transferred during the dissolution. A negative value of *E*_form_ indicates that the TAC is thermodynamically stable and experimentally accessible, while a positive value of *U*_diss_ signifies the electrochemical stability of the TAC. Figure 1c provides a graphical representation of the formation energy and dissolution potential of the examined TACs. The values for both *E*_form_ and *U*_diss_ can be found in Tables S1 and S2. We find that Ru_3_, Ag_3_, W_3_, Re_3_, Os_3_, Ir_3_, and Au_3_-TACs have high formation energy, suggesting they will be difficult to synthesize. We find that Sc_3_, Ti_3_, V_3_, Cr_3_, Mn_3_, Zn_3_, Y_3_, Zr_3_, Nb_3_, Mo_3_, Cd_3_, Hf_3_, and Ta_3_-TACs have negative *E*_form_ but negative *U*_diss_, suggesting that they can be synthesized but that they are not electrochemically stable. We find that seven homonuclear Fe_3_, Co_3_, Ni_3_, Cu_3_, Rh_3_, Pd_3_, and Pt_3_-TACs and all four bimetallic Fe_2_Ni, Co_2_Fe, Co_2_Ni, and Co_2_Cu-TACs have negative formation energies and positive dissolution potentials, suggesting they are synthetically feasible and electrochemically stable. Therefore, we considered only these 11 stable TACs (Fe_3_, Co_3_, Ni_3_, Cu_3_, Rh_3_, Pd_3_, Pt_3_, Fe_2_Ni, Co_2_Fe, Co_2_Ni, and Co_2_Cu-TACs) for the study of selectivity.

### 3.2 Selectivity of *NO_3_ and *H Adsorption on TACs

**Figure 2:**
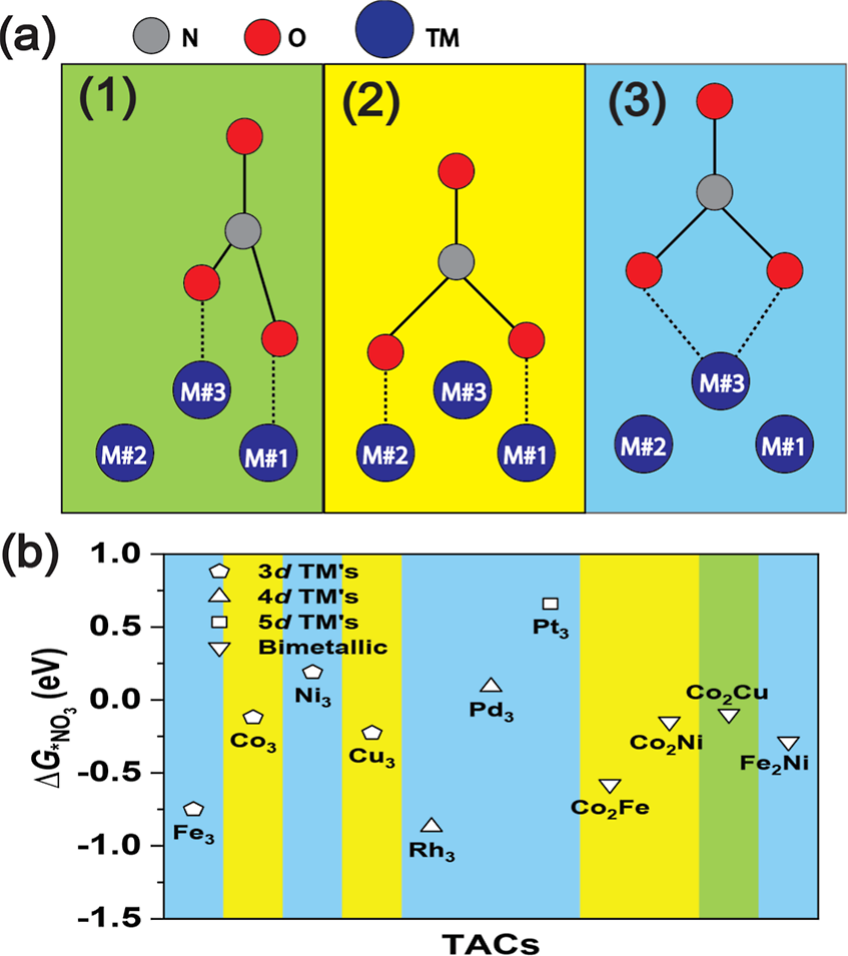
Nitrate adsorption configurations on the triple-atom site and respective adsorption free energies: (a) three potential NO_3_^–^ configurations on TAC surfaces. (b) NO_3_^–^ adsorption free energies on TAC surfaces and their associated configurations. The background colors in (b) indicate the adsorption configurations of *NO_3_ on respective TACs.

To kickstart the process of NO_3_RR, the first crucial step involves the absorption of the nitrate moiety. Figure 2a depicts the three different configurations of nitrate adsorption on the active site of TACs:

1 two oxygen atoms from the nitrate adsorbs on metal atoms M#1 or M#2 along with M#3 of the TAC;
2 two oxygen atoms from the nitrate adsorbs on metal atoms M#1 and M#2 of the TAC;
3 two oxygen atoms from the nitrate adsorbs on the metal atom M#3 atom of the TAC.

The adsorption free energy of a nitrate and its corresponding binding configuration on TACs are presented in Figure 2b. Configuration (1) represents the most stable adsorption state on Co_3_, Cu_3_, Co_2_Fe, Co_2_Ni. Configuration (2) represents the most stable adsorption state on Co_2_Cu. Configuration (3) represents the most stable adsorption state on Fe_3_, Ni_3_, Rh_3_, Pd_3_, Pt_3_, Fe_2_Ni. For Pt_3_-TAC, the adsorption of *NO_3_ is very weak, with ΔG_*NO_3__ of 0.66 eV. For the other 10 TACs, the adsorption of *NO_3_ is stronger, with ΔG_*NO_3__ varying from −0.87 to 0.19 eV. None of studied TACs adsorb nitrate too strongly (ΔG_*NO_3__< −3 eV), suggesting that these TACs would not be poisoned by nitrate during NO_3_RR. Values for the adsorption free energy of NO_3_^–^ for all possible configurations can be found in Table S3.

**Figure 3:**
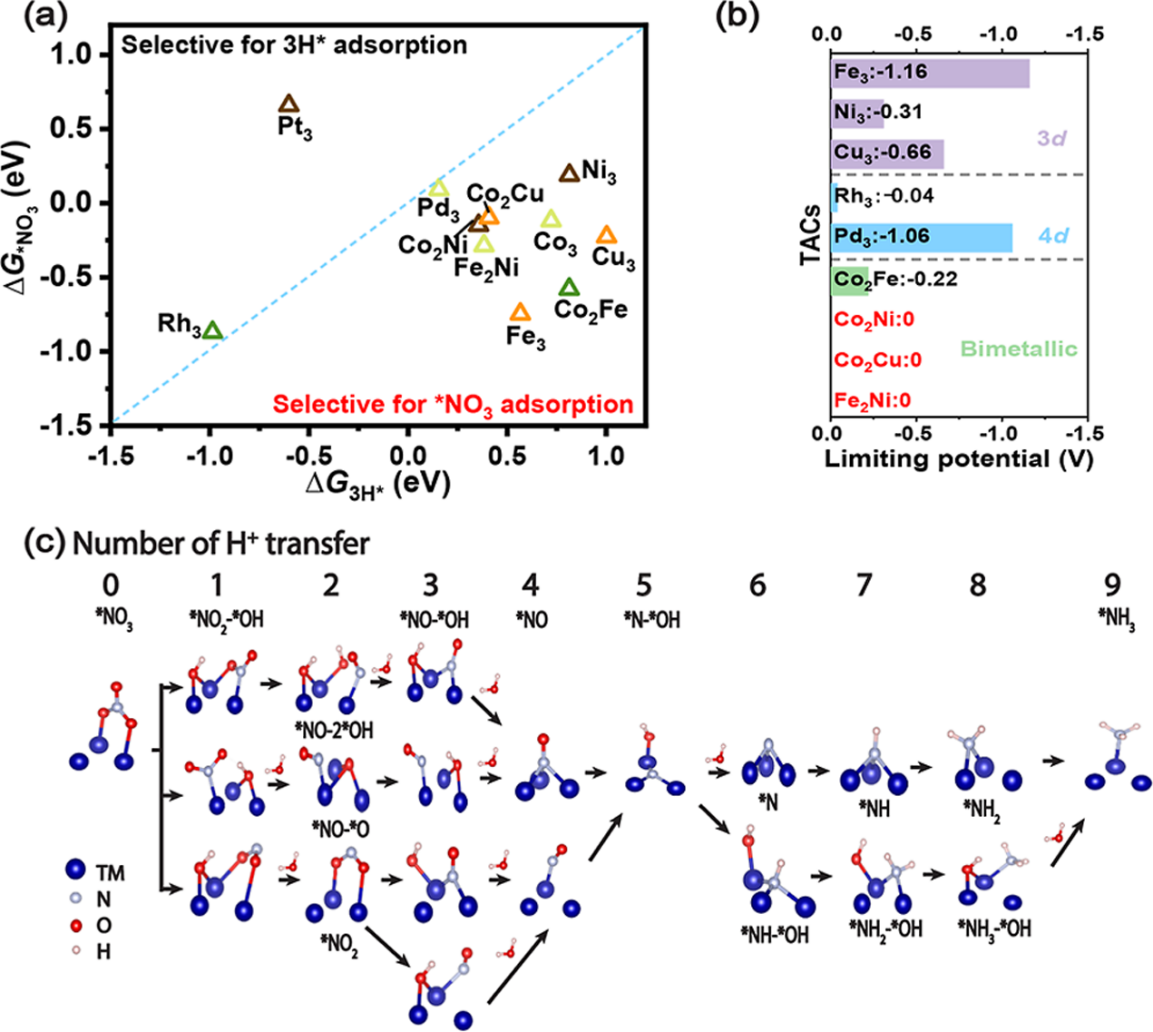
Electrochemical activity of TACs in nitrate-to-ammonia conversion. (a) Comparison of H and NO_3_^–^ adsorption energies on TACs. (b) Limiting potentials for NO_3_RR to NH_3_ on studied metal trimers. Ni_3_-TAC exhibits notable homonuclear electrocatalytic activity, with a limiting potential of −0.31 V. Meanwhile, Co_2_Ni-TAC, Co_2_Cu-TAC, and Fe_2_Ni-TAC show superior activity without potential-determining steps. (c) Schematic of observed NO_3_RR to NH_3_ mechanism on TACs.

Before delving into the details of the NO_3_RR mechanism on TACs, the competitive adsorption between hydrogen and nitrate should be studied.^8^ If the binding of *H is stronger than that of NO_3_^–^, NO_3_RR would be hindered due to poisoning of the active sites by hydrogen adsorption. Moreover, weak adsorption of nitrate may lead to the desorption of NO_3_^–^ from active sites before being reduced to NH_3_. Therefore, NO_3_RR would be highly preferable on catalysts that binds nitrate selectively rather than hydrogen. Figure 3a shows the adsorption free energy of 3*H versus *NO_3_ on TACs.^41^ Catalysts above the dashed line prefer binding to *H while ones below the dashed line prefer *NO_3_. For Pt_3_-TAC, the adsorption of *NO_3_ is much weaker than 3*H, with ΔG_*NO_3__ of 0.66 eV, indicating NO_3_RR is not likely to take place on Pt_3_-TAC. Thus, we considered only the other 10 TACs (Fe_3_, Co_3_, Ni_3_, Cu_3_, Rh_3_, Pd_3_, Fe_2_Ni, Co_2_Fe, Co_2_Ni, and Co_2_Cu-TACs) for studies of activity, excluding Pt_3_-TAC. However, Rh_3_-TAC and Pd_3_-TAC are located in or near the region selective for hydrogen adsorption, suggesting that the selectivity toward NO_3_RR is not very high; thus the FE on these two TACs would be lower compared with the other 8 TACs (Fe_3_, Co_3_, Ni_3_, Cu_3_, Fe_2_Ni, Co_2_Fe, Co_2_Ni, and Co_2_Cu-TACs).

### 3.3 NO_3_RR Mechanism on TACs

Reducing NO_3_^–^ to NH_3_ electrochemically at ambient conditions involves eight electron transfers and nine proton transfers, with the surplus due to the anionic nature of NO_3_^–^. The complexity arises from various intermediates and byproducts such as NO, NO_2_, and N_2_, posing a challenge for investigation.^6,8^ Furthermore, diverse adsorption configurations of reaction intermediates have been reported, indicating multiple binding possibilities for each intermediate^6,8^

We assessed the NO_3_RR electrochemical performance of the studied metal trimers based on the limiting potential (), where Δ*G*_max_ represents the maximum free energy change among all elementary steps that would be influenced by external potential. Although the overpotential is obtained as zero for some TACs, the rate-limiting step could either be the reaction barriers between each reaction intermediate or the desorption of ammonia, as ammonia desorption is not spontaneous. This evaluation culminated in the identification of the most favorable candidates for NH_3_ synthesis on M_3_-TACs, as detailed in Figure 3b. Here we are discussing reaction performance and mechanism at pH = 7. Notably, NH_3_ synthesis was most prominently observed on two homonuclear (Rh_3_ and Ni_3_) and four bimetallic (Co_2_Ni, Co_2_Cu, Fe_2_Ni, and Co_2_Fe) TACs, showing significantly lower limiting potentials compared to the others. Specifically, these six systems exhibit limiting potentials of −0.04, −0.31, 0.00, 0.00, 0.00, and −0.22 V respectively. Conversely, the Pd_3_ (−1.06 V) and Fe_3_ (−1.16 V) systems displayed unfavorable limiting potentials for effective NO_3_RR electrochemical performance.

Notably, although Rh_3_-TAC shows the lowest limiting potential among homonuclear TACs, we do not consider it as the best homonuclear NO_3_RR TAC due to both the low FE on Rh_3_-TAC, as discussed above in Section 3.2, and also because of the high cost and inappropriate oxidation state of precious Rh. The studied nitrogen-doped carbon is suitable to anchor metallic trimers with a total oxidation state of +6.^41^ However, the main oxidation state of a Rh atom is +3, suggesting that the Rh_3_ trimer may not fit into the structure. Co_3_-TAC is not regarded as a promising candidate for NO_3_RR, since its activity will be influenced by multiple nitrate adsorptions and preadsorption of H_2_O in the working environment, as shown in Table S5. In contrast, Ni_3_-TAC shows high selectivity and activity; its limiting potential will not be influenced by multiple nitrate adsorptions and preadsorption of *OH or H_2_O in the working environment; Ni is a nonprecious metal with a suitable oxidation state. Therefore, Ni_3_-TAC is regarded as the most promising homonuclear M_3_-TAC.

Compared to SACs or DACs, the synergy exhibited by metal trimers in TACs enables the accommodation of more intricate adsorption configurations and reaction pathways. Therefore, we conducted density functional theory (DFT) calculations to determine the Gibbs free energies for the elementary steps in nitrate reduction pathways toward NH_3_, aiming to explore the possible reaction pathways of NO_3_RR to NH_3_ on triple-atom sites. The NO_3_RR pathway is depicted by considering all plausible intermediates with the lowest total free energies. The observed NO_3_RR pathways on TACs are depicted in Figure 3c.

- The initial step is the adsorption of a nitrate ion onto the surface.
- Next is the protonation of the most accessible O atom accompanied by the breaking of a N–O bond, to form *NO_2_–*OH.
- In steps 3 to 5, three protons react with O atoms to release two water molecules, leaving *NO on the surface.
- Subsequently, in step 6, a proton attacks O atom in *NO accompanied by the breaking of the N–O bond, to form *N–*OH.
- In the 4 subsequent protonation steps 7 to 10, a molecule of ammonia and an additional water molecule form on the surface.

For all elementary steps observed during the search for minimum energy reaction pathways, the formulas are shown in Note S1.

**Figure 4:**
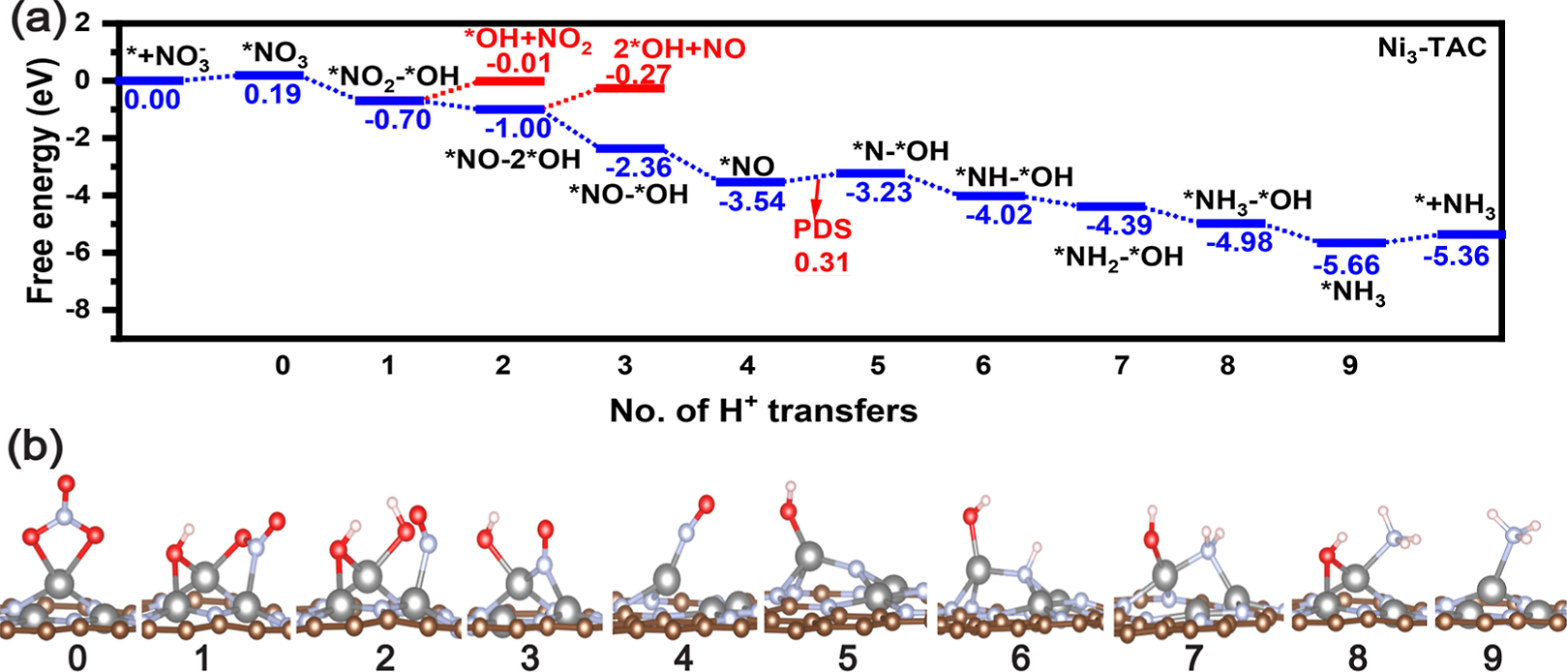
Reaction pathway (a) and corresponding optimized configurations (b) of NO_3_RR for Ni_3_-TAC.

**Figure 5:**
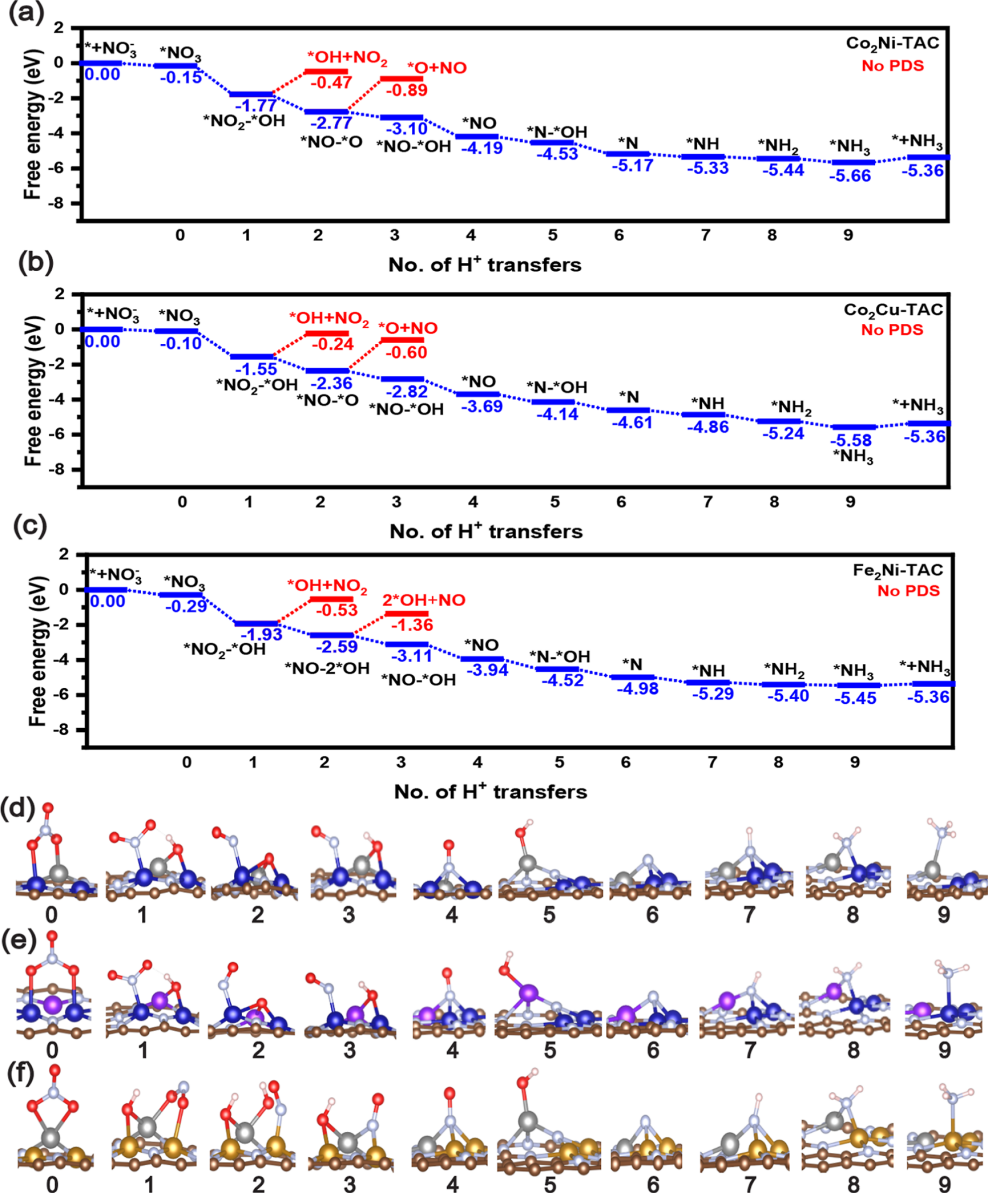
Reaction pathway of NO_3_RR for (a) Co_2_Ni-TAC, (b) Co_2_Cu-TAC, and (c) Fe_2_Ni-TAC. Corresponding optimized configurations of NO_3_RR for (d) Co_2_Ni-TAC, (e) Co_2_Cu-TAC, and (f) Fe_2_Ni-TAC.

To delve more deeply into the reaction mechanism and to assess the efficacy of nitrate reduction to NH_3_ on the M_3_-TACs, we conducted computations to determine the free energy profiles for NO_3_RR. We established the reference state as the bare surface at 0.0 eV. The free energy profiles and corresponding DFT-optimized structures for homonuclear Ni_3_-TAC are presented in Figure 4. Likewise, for bimetallic Co_2_Ni-TAC, Co_2_Cu-TAC, and Fe_2_Ni-TAC, the free energy profiles and relevant DFT-optimized structures can be found in Figure 5. The free energy profiles and their associated DFT-optimized structures for the remaining TACs are available in Figures S2–S7.

**Figure 6:**
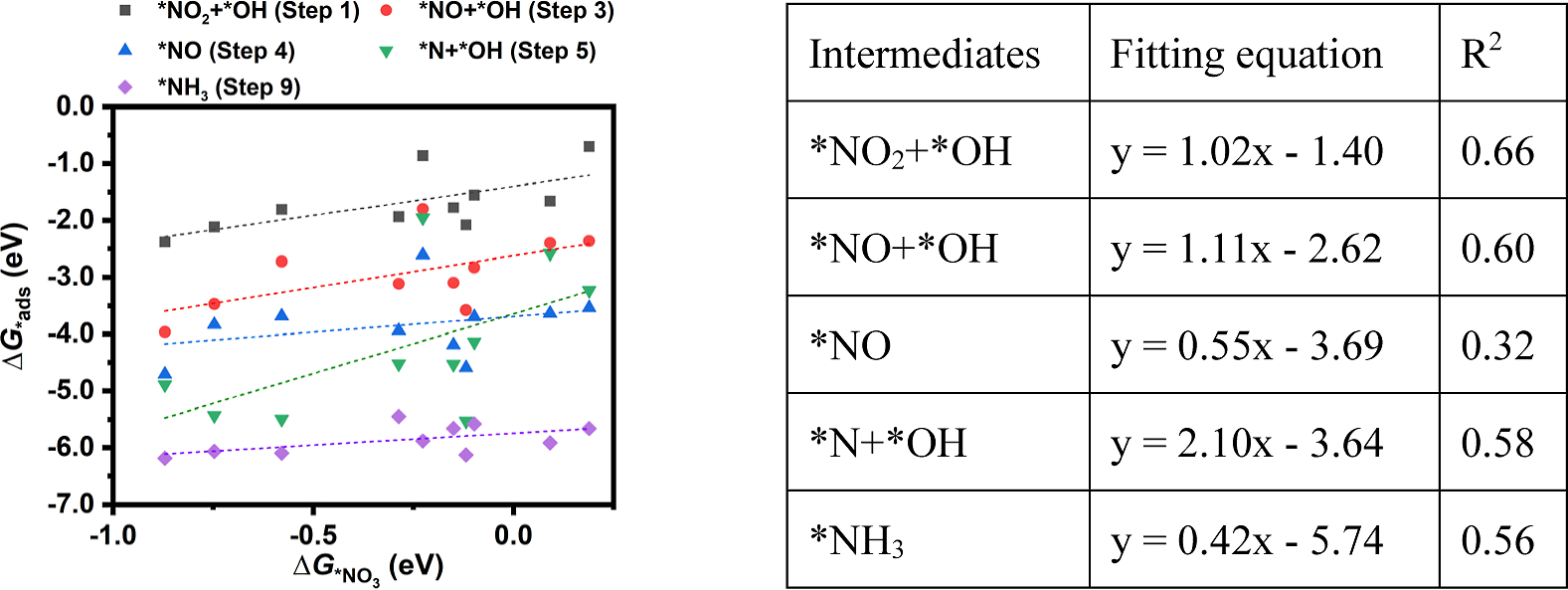
Adsorption free energies of intermediates in five protonation steps (step 1, step 3, step 4, step 5, and step 9) against ΔG_*NO_3__.

To investigate whether the adsorption of intermediates adheres to the scaling relationship, a pivotal challenge in developing high-performance single-atom electrocatalysts, we performed linear regression analysis for the adsorption free energies of intermediates in five protonation steps (step 1, step 3, step 4, step 5, and step 9) against ΔG_*NO_3__, as depicted in Figure 6. These five steps were chosen because in these steps, intermediates on all studied TACs are the same, although the adsorption configurations differ in some steps. Intermediates in these five steps are *NO_2_ + *OH, *NO + *OH, *NO, *N + *OH, and *NH_3_, separately. Remarkably, for all these steps, the adsorption free energies of the intermediates demonstrate a weak linear correlation with ΔG_*NO_3__, with *R*^2^ < 0.7, particularly in step 4 (*NO) with *R*^2^ = 0.33. This suggests that the design of TACs may serve as an effective approach to circumvent the scaling relationship among intermediates in NO_3_RR, which may facilitate the discovery of catalysts with ultrahigh activity.

To understand the advantage of TAC for NO_3_RR and the origin of breaking scaling relationship as discussed above, we analyzed the adsorption configurations of intermediates on all TACs along the NO_3_RR pathway. We found that for all these TACs, all three metal atoms participate in the adsorption of intermediates in many of the steps, although only one or two metal atoms contribute to the adsorption of *NO_3_ and *NH_3_. The collaborative function of metallic trimers enables the disruption of the scaling relationship that limits the efficacy of single-atom catalysts, in contrast to the conditions observed in SACs where intermediates can solely bind to a single active center. This suggests that the metallic trimers of TACs facilitate a smooth converting of nitrate to ammonia.

### 3.4 NO_3_RR Performance on Homonuclear TACs

The free energy profiles and corresponding DFT-optimized structures for Ni_3_-TAC are presented in Figure 4. Initially, two oxygen atoms in the nitrate moiety bind with Ni#3 to form *NO_3_, with a free energy change of 0.19 eV. The first step is the protonation of an O atom accompanied by breaking of a N–O bond in nitrate moiety, forming *NO_2_–*OH at −0.70 eV. In the optimized configuration of this step, *OH bonds with Ni#2 and Ni#3, while the N atom and an O atom in *NO_2_ bond with Ni#1 and Ni#3, respectively. The free energy of *OH + NO_2_ is −0.01 eV, indicating that the desorption of NO_2_ requires 0.69 eV, making it unfavorable. Next, a proton attacks the O in *NO_2_ that bonds with Ni#3, leading to breaking of an N–O bond to form *NO–2*OH at −1.00 eV. The free energy of 2*OH + NO is −0.27 eV, indicating the desorption of NO requires 0.73 eV, making it unfavorable. The two following downhill steps (*NO – 2*OH → *NO – *OH → *NO) are the protonation of two *OH to form two water molecules, leaving *NO bonding with three Ni atoms at −3.54 eV. Next is the protonation of O to form *N–*OH at −3.23 eV, with *OH bonding with Ni#3 and *N bonding with three Ni. Continuing, the sixth to eighth steps are consecutive protonation of N in intermediates (*N – *OH → *NH – *OH → *NH_2_ – *OH → *NH_3_ – *OH), with corresponding energy changes of −0.79, −0.37, and −0.59 eV, respectively. In the final step, a proton reacts with *OH exergonically to form a water molecule, leaving *NH_3_ at −5.66 eV. In the whole protonation process on Ni_3_-TAC, potential determining step is converting *NO to *N–*OH, requiring 0.31 eV.

For the Rh_3_-TAC case (as shown in Figure S2), the initial adsorption state of *NO_3_ and intermediates along the whole protonation pathway are the same as Ni_3_-TAC. In the whole protonation process on Rh_3_-TAC, the only endergonic step is converting *NH_3_–*OH to *NH_3_, requiring 0.04 eV. The desorption of *NO_2_ in *NO_2_–*OH and *NO in *NO–2*OH requires 1.48 and 1.35 eV, respectively, making them unfavorable. Although Rh_3_-TAC shows the lowest limiting potential among homonuclear TACs, we do not consider it as the best homonuclear NO_3_RR TAC due to the low FE on Rh_3_-TAC, as well as the high cost and inappropriate oxidation state of Rh, as discussed in 3.3.

Figure S3 presents the free energy profile and DFT-optimized structures for NO_3_RR for the Co_3_-TAC case without considering the influence of multiple nitrate adsorptions and preadsorption of H_2_O in the working environment, respectively. In the initial adsorption of the nitrate moiety, two oxygen atoms bind with Co#1 and Co#3, respectively, forming *NO_3_ at −0.12 eV. In the following five protonation steps (*NO_3_ → *NO_2_ – *OH → *NO – 2*OH → *NO – *OH → *NO → *N – *OH), intermediates in each step are the same as those on Ni_3_-TAC, with corresponding energy changes in each elementary step as −1.96, −0.77, −0.73, −1.01, and −0.97 eV, respectively. In the following step, the proton reacts with *OH to form a water molecule, leaving *N at −5.58 eV. Three subsequent steps are consecutive protonation of the nitrogen atom in intermediate steps to form an ammonia molecule (*N → *NH → *NH_2_ → *NH_3_), with corresponding energy changes of −0.31, 0.18, and −0.42 eV, respectively. In the whole protonation process on Co_3_-TAC, the only endergonic step is converting *NH to *NH_2_, requiring 0.18 eV. The desorption of *NO_2_ in *NO_2_–*OH and *NO in *NO–2*OH requires 1.37 and 1.70 eV, respectively, making them unfavorable.

For the Cu_3_-TAC case (as shown in Figure S4), intermediates in the first protonation step are also *NO_2_–*OH. Next is the protonation of *OH to form a water molecule, leaving *NO_2_ on the surface. In the following seven protonation steps, intermediates in each step are the same as those on Ni_3_-TAC. The potential determining step is converting *NO to form *N–*OH, with an energy change of 0.66 eV.

For the Pd_3_-TAC (as shown in Figure S5) and Fe_3_-TAC (as shown in Figure S6), intermediates along the whole protonation pathway are the same as those on Ni_3_-TAC. The potential determining step on Pd_3_-TAC is converting *NO to form *N–*OH, with an energy change of 1.06 eV. The potential determining step on Fe_3_-TAC (as shown in Figure S6) is converting *N–*OH to form *NH–*OH, with an energy change of 1.16 eV.

We compared the d-band centers and limiting potentials of Fe_3_, Ni_3_, and Cu_3_-TACs (as shown in Table S4), finding that the high activity of Ni_3_-TAC results from the medium energy level of its d-band center. For Fe_3_-TAC, its d-band center (−0.52 eV) is too high, and thus the adsorption of intermediates is too strong; for Cu_3_-TAC, the d-band center (−3.04 eV) is too low, and thus the adsorption of intermediates is too weak. Therefore, the NO_3_RR activity of Fe_3_ and Cu_3_ is low. By contrast, the d-band center of Ni_3_ (−1.48 eV) is located at medium energy level, leading to medium adsorption of intermediates and thus high activity.

### 3.5 NO_3_RR Performance on Bimetallic TACs

In the hope of discovering superior NO_3_RR catalysts, we further examined four bimetallic TACs by replacing a metal atom in homonuclear metallic trimers with a secondary metal atom. All studied bimetallic TACs are composed of Fe, Co, Ni, or Cu, because they are nonprecious metals with suitable oxidation states. Among the homonuclear nonprecious TACs without considering the influence of multiple nitrate adsorptions and preadsorption of H_2_O in the working environment, Co_3_-TAC shows the lowest limiting potential as discussed above, but it will be influenced by multiple nitrate adsorptions and preadsorption of H_2_O in the working environment. Therefore, we investigated three bimetallic Co_2_M-TACs (Co_2_Fe, Co_2_Ni, and Co_2_Cu) to test whether the secondary metal atom can enhance the performance of Co-based TACs. In addition, we studied Fe_2_Ni-TAC to test the possibility of improving Fe-based TACs by introducing a secondary metal atom.

To find the favorable position of the secondary metal atom, we compared the energy of each bimetallic TAC with the secondary metal atom at M#1 and M#3 (Figure S1), respectively, since the atomic environment of M#1 and M#2 are the same. We found that the bimetallic TAC with the secondary metal atom at M#3 has a lower energy than the corresponding TAC with the secondary metal atom at M#1, suggesting that a secondary metal atom at M#3 is energetically preferable for bimetallic TACs. Therefore, we studied the stability, selectivity, and activity of TACs with the secondary metal atom at M#3. Since the stability and selectivity of these bimetallic TACs are favorable for NO_3_RR, as discussed above, we only analyze the activity below.

For Co_2_Ni-TAC (as shown in Figure 5a,d) and Co_2_Cu-TAC (as shown in Figure 5b,e), the reaction pathway is the same. The first step is protonation of the most accessible O atom in *NO_3_, forming *NO_2_–*OH with *NO_2_ bonding to Co#2 and *OH bonding with Co#1 and the secondary metal atom. In the second step, a proton attacks an O atom in *NO_2_, accompanied by the breaking of an N–O bond in *NO_2_ and an H–O bond in *OH to form a water molecule, leaving *NO–*O on the surface. Next is the protonation of *O to form *NO–*OH, the same intermediates as the third protonation step on Co_3_-TAC. For the following six protonation steps, the intermediates are the same as those on Co_3_-TAC. For Co_2_Ni-TAC, the desorption of *NO_2_ in *NO_2_–*OH and *NO in *NO–*O requires 1.30 and 1.88 eV, respectively, making them unfavorable. For Co_2_Cu-TAC, it requires 1.31 and 1.76 eV, respectively, making them unfavorable. Notably, the whole protonation pathway on Co_2_Ni-TAC and Co_2_Cu-TAC is downhill, suggesting that they are superior NO_3_RR catalysts with no potential determining step. Of course, in our current calculations, we do not consider activation barriers so kinetic factors are not considered. Thus, in realistic conditions, small overpotentials will be needed to drive the NO_3_RR on Co_2_Ni-TAC and Co_2_Cu-TAC.

For Fe_2_Ni-TAC (as shown in Figure 5c,f), intermediates in the whole reaction pathway are the same as those on Co_3_-TAC. The desorption of *NO_2_ in *NO_2_–*OH and *NO in *NO–*2OH require 1.40 and 2.23 eV, respectively, making them unfavorable. Notably, the whole protonation pathway on Fe_2_Ni-TAC is also downhill, just as for Co_2_Ni-TAC and Co_2_Cu-TAC discussed above, suggesting that they are superior NO_3_RR catalysts with no potential determining step. As discussed above, in realistic conditions, small overpotentials will be needed to drive the NO_3_RR on these three TACs.

For Co_2_Fe-TAC (as shown in Figure S7), intermediates in the whole reaction pathway are the same as those on Fe_3_-TAC. The desorption of *NO_2_ in *NO_2_–*OH and *NO in *NO–*2OH requires 1.21 and 1.55 eV, respectively, making them unfavorable. The potential determining step on Co_2_Fe-TAC is converting *N–*OH to form *NH–*OH, with an energy change of 0.22 eV.

The comparison of the electrochemical performance of the electrocatalysts and their potential-determining steps are listed in Table 1. Bimetallic Co_2_Ni@NC, Co_2_Cu@NC, and Fe_2_Ni@NC TACs show theoretical limiting potentials of 0.00 *V*_RHE_, representing the best theoretical activity been reported up to date.

**Table 1:** Comparison of Electrocatalytic Performance of NO_3_RR on Atomically Dispersed Metal Catalysts Supported by 2D Substrates

catalyst	potential-determining step	limiting potential (V)	reference
Co_2_Ni@NC	none	0.00	
Co_2_Cu@NC	none	0.00	
Fe_2_Ni@NC	none	0.00	this work
Co_2_Fe@NC	*N – *OH → *NH – *OH	–0.22	
Ni_3_@NC	*NO → *N – *OH	–0.31	
Ru/g-C_3_N_4_	*NO → *NOH	–0.34	(54)
Ti/g-CN	*NO → *NOH	–0.39	(6)
Os–N_4_/C	*N → *NH	–0.42	(55)
Pt–N_4_/C	*NO → *NOH	–0.48	(56)
Cr_2_-Pc	*O_2_H_3_ → 2H_2_O	–0.02	(8)
FeMo@g-CN	*NO → *NOH	–0.34	(9)
Cu_2_@NC	*NO_3_ → *NO_3_H	–0.36	(57)

**Figure 7:**
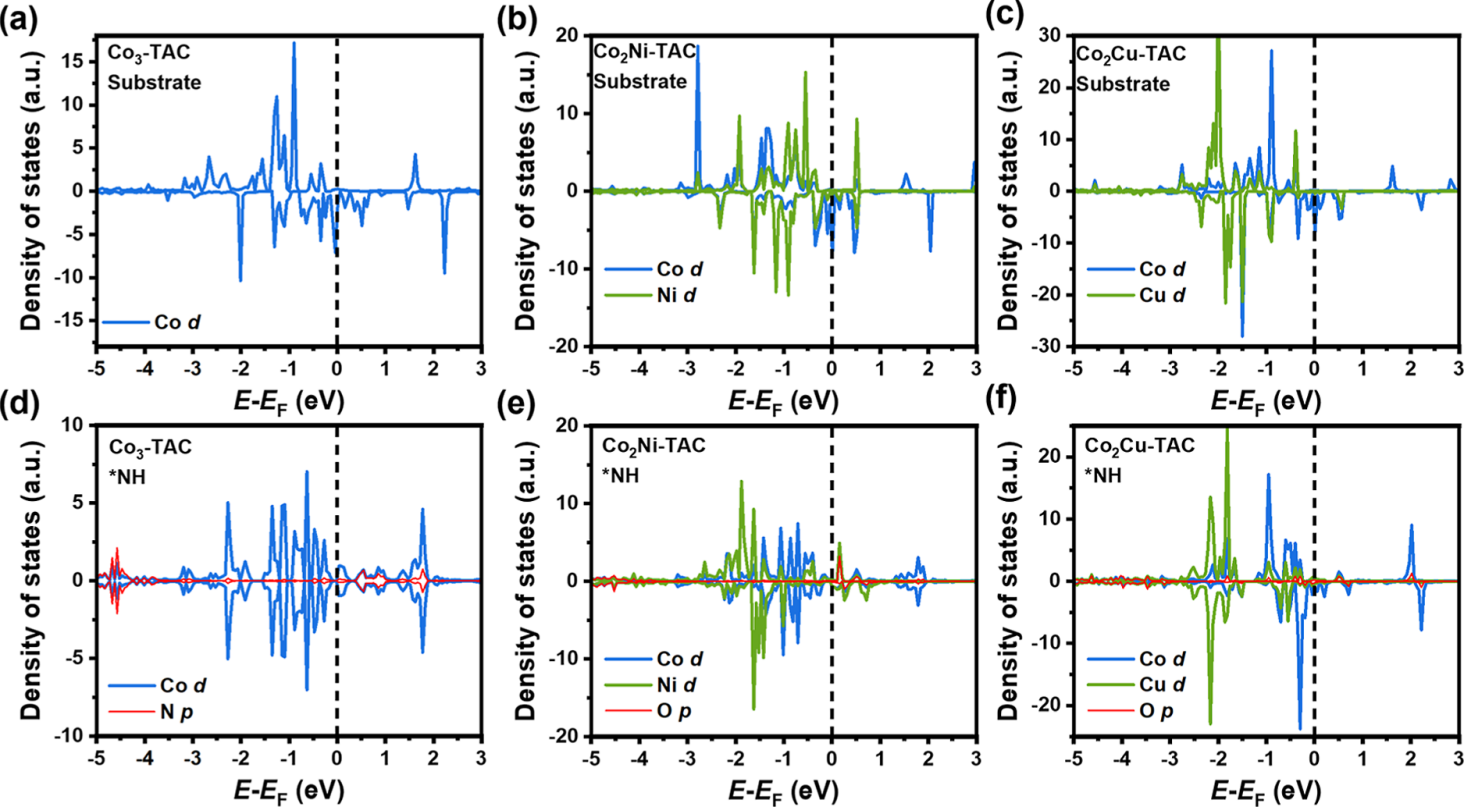
Electronic structures of Co_3_-TAC, Co_2_Ni-TAC, and Co_2_Cu-TAC. The partial density of states (PDOS) of Co_3_-TAC before (a) and after (d) the adsorption of *NH intermediate. PDOS of Co_2_Ni-TAC before (b) and after (e) the adsorption of *NH intermediate. PDOS of Co_2_Cu-TAC before (c) and after (f) the adsorption of *NH intermediate. The Fermi levels are indicated by the black dash lines.

To understand the electronic impact of the secondary metal atom, we compared the projected density of states (PDOS) of related atomic orbitals on Co_3_-TAC, Co_2_Ni-TAC, and Co_2_Cu-TAC without and with adsorption of the *NH intermediate, as shown in Figure 7. We chose to analyze *NH based on the following considerations. Although the adsorption configuration of *NH is similar on three TACs, too strong adsorption of *NH on Co_3_-TAC contributes to the potential determining step, while relatively weak adsorption of *NH on Co_2_Ni-TAC and Co_2_Cu-TAC facilitates the smooth NO_3_RR without potential determining step. This indicates that compared with homonuclear Co_3_-TAC, the secondary Ni or Cu weakens the adsorption of *NH on Co-based TACs. By comparing the PDOS of d orbital of Co#2 in three TACs without *NH, as shown in Figure 7a–c, we found that the shape of the PDOS differs in the three conditions, suggesting that the secondary Ni and Cu influence the electron states of Co atoms. By comparing the PDOS of the d orbital of Co#2 and the p orbital of N in *NH for the three TACs (Figure 7d–f), we found that the N peaks overlap with the peaks of Co, indicating that *NH binds with Co atoms in the three TACs. By comparing the PDOS of the d orbital of the secondary Ni or Cu and the p orbital of N in *NH for bimetallic TACs (Figure 7e–f), we found that the peaks of N overlap with the peaks of the secondary atom, indicating that *NH binds to the secondary atoms in bimetallic TACs. Therefore, the secondary Ni and Cu atoms not only influence the electron states of Co atoms in bimetallic Co_2_Ni and Co_2_Cu TACs, but also bond with reaction intermediates.

Both the secondary metal elements and Co bind with *NH intermediates (as shown in the adsorption configuration in Figure 5d,e, as well as the PDOS in Figure 7e,f). Compared with Co_3_-TAC (ΔG_*NH_ = −5.89 eV in Figure S3a), we can find that the Ni (ΔG_*NH_ = −5.33 eV of Co_2_Ni-TAC in Figure 5a) or Cu replacement (ΔG_*NH_ = −4.86 eV of Co_2_Cu-TAC in Figure 5b) weakens the binding strength between Co and *NH. Besides, the weakening effect is also indicated by the PDOS. The p orbital of the N atom forms strong bonding with the d orbital of the Co atom in Co_3_-TAC around −4.5 eV below Fermi level (shown in Figure 7d), while only weak peaks appear in Co_2_Ni-TAC (shown in Figure 7e) and Co_2_Cu-TAC (shown in Figure 7f).

## 4 Conclusions

In summary, Quantum Mechanics calculations were utilized to evaluate the potential of TACs supported on nitrogen-doped carbon for nitrate reduction to ammonia. By studying the reaction pathways, we found five highly active nonprecious candidates with significantly low limiting potentials. Four of them are bimetallic TACs: Co_2_Ni (0.00 V), Co_2_Cu (0.00 V), Fe_2_Ni (0.00 V), and Co_2_Fe (−0.22 V). And the other one is homonuclear Ni_3_-TAC (−0.31 V). We predicted that these catalysts show high selectivity for converting NO_3_^–^ to NH_3_, due to stronger adsorption of nitrate than hydrogen, and high energy barriers for releasing byproducts (NO_2_ and NO). By analyzing the density of states, we find that in bimetallic Co_2_Ni and Co_2_Cu-TACs, the secondary Ni and Cu atoms not only bond with intermediates, but also influence the electron states of Co atoms. We find that the collaboration among three active sites can disrupt the scaling relationship among intermediates in NO_3_RR, resulting in catalysts boasting remarkably high activity. This heralds a new era in the development of efficient electrocatalysts for nitrate reduction and ammonia synthesis based on the triple-atom platform.

## Data Availability

Data will be available on request.

## Supporting Information Available

Formula of all elementary process observed during the search of minimum energy reaction pathways. During structural relaxation, three carbon atoms in each TAC model are fixed to reduce the distortion and corresponding energy error (PDF)

## References

[ref1] Gruber N.; Galloway J. N. An Earth-System Perspective of the Global Nitrogen Cycle. Nature 2008, 451 (7176), 293–296. 10.1038/nature06592. 18202647

[ref2] Canfield D. E.; Glazer A. N.; Falkowski P. G. The Evolution and Future of Earth’s Nitrogen Cycle. Science 2010, 330 (6001), 192–196. 10.1126/science.1186120. 20929768

[ref3] Chen J. G.; Crooks R. M.; Seefeldt L. C.; Bren K. L.; Bullock R. M.; Darensbourg M. Y.; Holland P. L.; Hoffman B.; Janik M. J.; Jones A. K.; Kanatzidis M. G.; King P.; Lancaster K. M.; Lymar S. V.; Pfromm P.; Schneider W. F.; Schrock R. R. Beyond Fossil Fuel–Driven Nitrogen Transformations. Science 2018, 360 (6391), eaar6611 10.1126/science.aar6611. 29798857 PMC6088796

[ref4] Butcher D. P.; Gewirth A. A. Nitrate Reduction Pathways on Cu Single Crystal Surfaces: Effect of Oxide and Cl. Nano Energy 2016, 29, 457–465. 10.1016/j.nanoen.2016.06.024.

[ref5] Gao X.; Tse E. C. M. Unraveling the Performance Descriptors for Designing Single-Atom Catalysts on Defective MXenes for Exclusive Nitrate-To-Ammonia Electrocatalytic Upcycling. Small 2024, 20 (11), 2306311 10.1002/smll.202306311. 37936311

[ref6] Niu H.; Zhang Z.; Wang X.; Wan X.; Shao C.; Guo Y. Theoretical Insights into the Mechanism of Selective Nitrate-to-Ammonia Electroreduction on Single-Atom Catalysts. Adv. Funct. Mater. 2021, 31 (11), 2008533 10.1002/adfm.202008533.

[ref7] Zhao T.; Chen K.; Xu X.; Li X.; Zhao X.; Cai Q.; Chu K.; Zhao J. Homonuclear Dual-Atom Catalysts Embedded on N-Doped Graphene for Highly Efficient Nitrate Reduction to Ammonia: From Theoretical Prediction to Experimental Validation. Appl. Catal., B 2023, 339, 123156 10.1016/j.apcatb.2023.123156.

[ref8] Rehman F.; Kwon S.; Musgrave C. B.; Tamtaji M.; Goddard W. A.; Luo Z. High-Throughput Screening to Predict Highly Active Dual-Atom Catalysts for Electrocatalytic Reduction of Nitrate to Ammonia. Nano Energy 2022, 103, 107866 10.1016/j.nanoen.2022.107866.

[ref9] Shu Z.; Chen H.; Liu X.; Jia H.; Yan H.; Cai Y. High-Throughput Screening of Heterogeneous Transition Metal Dual-Atom Catalysts by Synergistic Effect for Nitrate Reduction to Ammonia. Adv. Funct. Mater. 2023, 33 (32), 2301493 10.1002/adfm.202301493.

[ref10] Luo F.; Guo L. Bimetallic Synergistic Catalysts Based on Two-Dimensional Carbon-Rich Conjugated Frameworks for Nitrate Electrocatalytic Reduction to Ammonia: Catalyst Screening and Mechanism Insights. Nanotechnology 2024, 35 (12), 125201 10.1088/1361-6528/ad1649. 38100833

[ref11] Haber F.; Le Rossignol R. Über Die Technische Darstellung von Ammoniak Aus Den Elementen. Z. Elektrochem. Angew. Phys. Chem. 1913, 19 (2), 53–72. 10.1002/bbpc.19130190201.

[ref12] Kandemir T.; Schuster M. E.; Senyshyn A.; Behrens M.; Schlögl R. The Haber–Bosch Process Revisited: On the Real Structure and Stability of “Ammonia Iron” under Working Conditions. Angew. Chem., Int. Ed. 2013, 52 (48), 12723–12726. 10.1002/anie.201305812. 24123652

[ref13] Hirakawa H.; Hashimoto M.; Shiraishi Y.; Hirai T. Selective Nitrate-to-Ammonia Transformation on Surface Defects of Titanium Dioxide Photocatalysts. ACS Catal. 2017, 7 (5), 3713–3720. 10.1021/acscatal.7b00611.

[ref14] Rosca V.; Duca M.; de Groot M. T.; Koper M. T. M. Nitrogen Cycle Electrocatalysis. Chem. Rev. 2009, 109 (6), 2209–2244. 10.1021/cr8003696. 19438198

[ref15] Zheng X.; Yan Y.; Li X.; Liu Y.; Yao Y. Theoretical Insights into Dissociative-Associative Mechanism for Enhanced Electrochemical Nitrate Reduction to Ammonia. J. Hazard. Mater. 2023, 446, 130679 10.1016/j.jhazmat.2022.130679. 36580786

[ref16] Yang L.; Feng S.; Zhu W. Achieving Reaction Pathway Separation for Electrochemical Nitrate Fixation on Triatomic Catalysts: A New Mechanism. J. Hazard. Mater. 2023, 441, 129972 10.1016/j.jhazmat.2022.129972.

[ref17] Fang L.; Wang S.; Song C.; Yang X.; Li Y.; Liu H. Enhanced Nitrate Reduction Reaction via Efficient Intermediate Nitrite Conversion on Tunable CuxNiy/NC Electrocatalysts. J. Hazard. Mater. 2022, 421, 126628 10.1016/j.jhazmat.2021.126628. 34343879

[ref18] Wang A.; Li J.; Zhang T. Heterogeneous Single-Atom Catalysis. Nat. Rev. Chem 2018, 2 (6), 65–81. 10.1038/s41570-018-0010-1.

[ref19] Mitchell S.; Pérez-Ramírez J. Single Atom Catalysis: A Decade of Stunning Progress and the Promise for a Bright Future. Nat. Commun. 2020, 11 (1), 4302 10.1038/s41467-020-18182-5. 32855411 PMC7453014

[ref20] Zhu C.; Fu S.; Shi Q.; Du D.; Lin Y. Single-Atom Electrocatalysts. Angew. Chem., Int. Ed. 2017, 56 (45), 13944–13960. 10.1002/anie.201703864. 28544221

[ref21] Wang Y.; Wang D.; Li Y. Rational Design of Single-Atom Site Electrocatalysts: From Theoretical Understandings to Practical Applications. Adv. Mater. 2021, 33 (34), 2008151 10.1002/adma.202008151. 34240475

[ref22] Zhang Q.; Guan J. Single-Atom Catalysts for Electrocatalytic Applications. Adv. Funct. Mater. 2020, 30 (31), 2000768 10.1002/adfm.202000768.

[ref23] Tamtaji M.; Kim M. G.; Wang J.; Galligan P. R.; Zhu H.; Hung F.-F.; Xu Z.; Zhu Y.; Luo Z.; Goddard W. A.; Chen G. A High-Entropy Single-Atom Catalyst Toward Oxygen Reduction Reaction in Acidic and Alkaline Conditions. Advanced Science 2024, 11 (26), 2309883 10.1002/advs.202309883. 38687196 PMC11234427

[ref24] Li R.; Wang D. Superiority of Dual-Atom Catalysts in Electrocatalysis: One Step Further Than Single-Atom Catalysts. Adv. Energy Mater. 2022, 12 (9), 2103564 10.1002/aenm.202103564.

[ref25] Tamtaji M.; Kim M. G.; Li Z.; Cai S.; Wang J.; Galligan P. R.; Hung F.-F.; Guo H.; Chen S.; Luo Z.; Wu W.; Goddard W. A.; Chen G. High-Throughput Screening of Dual Atom Catalysts for Oxygen Reduction and Evolution Reactions and Rechargeable Zinc-Air Battery. Nano Energy 2024, 126, 109634 10.1016/j.nanoen.2024.109634.

[ref26] Lin X.; Li Q.; Hu Y.; Jin Z.; Reddy K. M.; Li K.; Lin X.; Ci L.; Qiu H.-J. Revealing Atomic Configuration and Synergistic Interaction of Single-Atom-Based Zn-Co-Fe Trimetallic Sites for Enhancing Oxygen Reduction and Evolution Reactions. Small 2023, 19 (30), 2300612 10.1002/smll.202300612. 37058090

[ref27] Ji S.; Chen Y.; Fu Q.; Chen Y.; Dong J.; Chen W.; Li Z.; Wang Y.; Gu L.; He W.; Chen C.; Peng Q.; Huang Y.; Duan X.; Wang D.; Draxl C.; Li Y. Confined Pyrolysis within Metal–Organic Frameworks To Form Uniform Ru3 Clusters for Efficient Oxidation of Alcohols. J. Am. Chem. Soc. 2017, 139 (29), 9795–9798. 10.1021/jacs.7b05018. 28696113

[ref28] Xiao J.; Liu Z.; Wang X.; Li F.; Zhao Z. Homonuclear Multi-Atom Catalysts for CO2 Electroreduction: A Comparison Density Functional Theory Study with Their Single-Atom Counterparts. J. Mater. Chem. A 2023, 11 (46), 25662–25670. 10.1039/D3TA05498E.

[ref29] Chen Z. W.; Chen L. X.; Jiang M.; Chen D.; Wang Z. L.; Yao X.; Singh C. V.; Jiang Q. A Triple Atom Catalyst with Ultrahigh Loading Potential for Nitrogen Electrochemical Reduction. J. Mater. Chem. A 2020, 8 (30), 15086–15093. 10.1039/D0TA04919K.

[ref30] Chen Z. W.; Chen L. X.; Yang C. C.; Jiang Q. Atomic (Single, Double, and Triple Atoms) Catalysis: Frontiers, Opportunities, and Challenges. J. Mater. Chem. A 2019, 7 (8), 3492–3515. 10.1039/C8TA11416A.

[ref31] Cai G.; Lv H.; Zhang G.; Liu D.; Zhang J.; Zhu J.; Xu J.; Kong X.; Jin S.; Wu X.; Ji H. A Volcano Correlation between Catalytic Activity for Sulfur Reduction Reaction and Fe Atom Count in Metal Center. J. Am. Chem. Soc. 2024, 146, 13055 10.1021/jacs.3c14312. 38695850

[ref32] Shang H.; Zhou X.; Dong J.; Li A.; Zhao X.; Liu Q.; Lin Y.; Pei J.; Li Z.; Jiang Z.; Zhou D.; Zheng L.; Wang Y.; Zhou J.; Yang Z.; Cao R.; Sarangi R.; Sun T.; Yang X.; Zheng X.; Yan W.; Zhuang Z.; Li J.; Chen W.; Wang D.; Zhang J.; Li Y. Engineering Unsymmetrically Coordinated Cu-S1N3 Single Atom Sites with Enhanced Oxygen Reduction Activity. Nat. Commun. 2020, 11 (1), 3049 10.1038/s41467-020-16848-8. 32546781 PMC7297793

[ref33] Peng L.; Shang L.; Zhang T.; Waterhouse G. I. N. Recent Advances in the Development of Single-Atom Catalysts for Oxygen Electrocatalysis and Zinc–Air Batteries. Adv. Energy Mater. 2020, 10 (48), 2003018 10.1002/aenm.202003018.

[ref34] Wan C.; Duan X.; Huang Y. Molecular Design of Single-Atom Catalysts for Oxygen Reduction Reaction. Adv. Energy Mater. 2020, 10 (14), 1903815 10.1002/aenm.201903815.

[ref35] Zhang J.; Yang H.; Liu B. Coordination Engineering of Single-Atom Catalysts for the Oxygen Reduction Reaction: A Review. Adv. Energy Mater. 2021, 11 (3), 2002473 10.1002/aenm.202002473.

[ref36] Li Y.; Feng Y.; Zheng D.; Zhao X.; Zhou Y.; Fu X.; Chen X. High-Performance Screening of Carbon-Nitride Single-Atom Catalysts for Oxygen Electrode Reaction in Rechargeable Metal–Air Batteries. Chem. Eng. J. 2023, 476, 146753 10.1016/j.cej.2023.146753.

[ref37] Pang Y.; Su C.; Xu L.; Shao Z. When Nitrogen Reduction Meets Single-Atom Catalysts. Prog. Mater. Sci. 2023, 132, 101044 10.1016/j.pmatsci.2022.101044.

[ref38] Li M.; Wang H.; Luo W.; Sherrell P. C.; Chen J.; Yang J. Heterogeneous Single-Atom Catalysts for Electrochemical CO2 Reduction Reaction. Adv. Mater. 2020, 32 (34), 2001848 10.1002/adma.202001848. 32644259

[ref39] Wang T.; Zhao Q.; Fu Y.; Lei C.; Yang B.; Li Z.; Lei L.; Wu G.; Hou Y. Carbon-Rich Nonprecious Metal Single Atom Electrocatalysts for CO2 Reduction and Hydrogen Evolution. Small Methods 2019, 3 (10), 1900210 10.1002/smtd.201900210.

[ref40] Wang Y.; Liu Y.; Liu W.; Wu J.; Li Q.; Feng Q.; Chen Z.; Xiong X.; Wang D.; Lei Y. Regulating the Coordination Structure of Metal Single Atoms for Efficient Electrocatalytic CO2 Reduction. Energy Environ. Sci. 2020, 13 (12), 4609–4624. 10.1039/D0EE02833A.

[ref41] Tamtaji M.; Kwon S.; Musgrave C. B. I. I. I.; Goddard W. A. I. I. I.; Chen G. Reaction Mechanism of Rapid CO Electroreduction to Propylene and Cyclopropane (C3+) over Triple Atom Catalysts. ACS Appl. Mater. Interfaces 2024, 16, 50567 10.1021/acsami.4c06257. 38919050

[ref42] Yang H.; Zou W.; Zhang C.; Du A. Ab Initio Studies of Electrocatalytic CO2 Reduction for Small Cu Cluster Supported on Polar Substrates. ACS Appl. Mater. Interfaces 2024, 16 (26), 33688–33695. 10.1021/acsami.4c07445. 38900983

[ref43] Perdew J. P.; Burke K.; Ernzerhof M. Generalized Gradient Approximation Made Simple. Phys. Rev. Lett. 1996, 77 (18), 3865–3868. 10.1103/PhysRevLett.77.3865. 10062328

[ref44] Kresse G.; Furthmüller J. Efficiency of Ab-Initio Total Energy Calculations for Metals and Semiconductors Using a Plane-Wave Basis Set. Comput. Mater. Sci. 1996, 6 (1), 15–50. 10.1016/0927-0256(96)00008-0.

[ref45] Kresse G.; Furthmüller J. Efficient Iterative Schemes for Ab Initio Total-Energy Calculations Using a Plane-Wave Basis Set. Phys. Rev. B 1996, 54 (16), 11169–11186. 10.1103/PhysRevB.54.11169. 9984901

[ref46] Kresse G.; Joubert D. From Ultrasoft Pseudopotentials to the Projector Augmented-Wave Method. Phys. Rev. B 1999, 59 (3), 1758–1775. 10.1103/PhysRevB.59.1758.

[ref47] Grimme S. Semiempirical GGA-Type Density Functional Constructed with a Long-Range Dispersion Correction. J. Comput. Chem. 2006, 27 (15), 1787–1799. 10.1002/jcc.20495. 16955487

[ref48] Grimme S.; Antony J.; Ehrlich S.; Krieg H. A Consistent and Accurate Ab Initio Parametrization of Density Functional Dispersion Correction (DFT-D) for the 94 Elements H-Pu. J. Chem. Phys. 2010, 132 (15), 154104 10.1063/1.3382344. 20423165

[ref49] Mathew K.; Sundararaman R.; Letchworth-Weaver K.; Arias T. A.; Hennig R. G. Implicit Solvation Model for Density-Functional Study of Nanocrystal Surfaces and Reaction Pathways. J. Chem. Phys. 2014, 140 (8), 084106 10.1063/1.4865107. 24588147

[ref50] Mathew K.; Kolluru V. S. C.; Mula S.; Steinmann S. N.; Hennig R. G. Implicit Self-Consistent Electrolyte Model in Plane-Wave Density-Functional Theory. J. Chem. Phys. 2019, 151 (23), 234101 10.1063/1.5132354. 31864239

[ref51] Nørskov J. K.; Rossmeisl J.; Logadottir A.; Lindqvist L.; Kitchin J. R.; Bligaard T.; Jónsson H. Origin of the Overpotential for Oxygen Reduction at a Fuel-Cell Cathode. J. Phys. Chem. B 2004, 108 (46), 17886–17892. 10.1021/jp047349j. 39682080

[ref52] Cai G.; Lv H.; Zhang G.; Liu D.; Zhang J.; Zhu J.; Xu J.; Kong X.; Jin S.; Wu X.; Ji H. A Volcano Correlation between Catalytic Activity for Sulfur Reduction Reaction and Fe Atom Count in Metal Center. J. Am. Chem. Soc. 2024, 146 (19), 13055–13065. 10.1021/jacs.3c14312. 38695850

[ref53] Fang C.; Zhou J.; Zhang L.; Wan W.; Ding Y.; Sun X. Synergy of Dual-Atom Catalysts Deviated from the Scaling Relationship for Oxygen Evolution Reaction. Nat. Commun. 2023, 14 (1), 4449 10.1038/s41467-023-40177-1. 37488102 PMC10366111

[ref54] Lv L.; Shen Y.; Liu J.; Meng X.; Gao X.; Zhou M.; Zhang Y.; Gong D.; Zheng Y.; Zhou Z. Computational Screening of High Activity and Selectivity TM/g-C3N4 Single-Atom Catalysts for Electrocatalytic Reduction of Nitrates to Ammonia. J. Phys. Chem. Lett. 2021, 12 (45), 11143–11150. 10.1021/acs.jpclett.1c03005. 34756048

[ref55] Wang S.; Gao H.; Li L.; Hui K. S.; Dinh D. A.; Wu S.; Kumar S.; Chen F.; Shao Z.; Hui K. N. High-Throughput Identification of Highly Active and Selective Single-Atom Catalysts for Electrochemical Ammonia Synthesis through Nitrate Reduction. Nano Energy 2022, 100, 107517 10.1016/j.nanoen.2022.107517.

[ref56] Wang Y.; Shao M. Theoretical Screening of Transition Metal–N4-Doped Graphene for Electroreduction of Nitrate. ACS Catal. 2022, 12 (9), 5407–5415. 10.1021/acscatal.2c00307.

[ref57] Lv P.; Wu D.; He B.; Li X.; Zhu R.; Tang G.; Lu Z.; Ma D.; Jia Y. An Efficient Screening Strategy towards Multifunctional Catalysts for the Simultaneous Electroreduction of NO3–, NO2– and NO to NH3. J. Mater. Chem. A 2022, 10 (17), 9707–9716. 10.1039/D2TA00192F.

