# Supplementary material for: Nonprecious Triple-Atom Catalysts with Ultrahigh Activity for Electrochemical Reduction of Nitrate to Ammonia: A DFT Screening
Source: ACS Appl Mater Interfaces. 2025 Jan 10;17(3):4854–64. doi: 10.1021/acsami.4c17726 (PMC11803552; doi:10.1021/acsami.4c17726)
Supplement: Supplementary file 1 — am4c17726_si_001.pdf [file am4c17726_si_001.pdf]

# Supporting Information

# Non-precious triple-atom catalysts with ultra-high activity for electrochemical reduction of nitrate to ammonia: a DFT screening

Xiangyi Zhou[1†], Mohsen Tamtaji[2†], Weijun Zhou[3], William A. Goddard III[4*], GuanHua Chen[1,2*]

*[1]Department of Chemistry, The University of Hong Kong, Pokfulam Road, Hong Kong SAR, 999077, China*

*[2]Hong Kong Quantum AI Lab Limited, Pak Shek Kok, Hong Kong SAR, 999077, China*

*[3]QuantumFabless Limited, Pak Shek Kok, Hong Kong SAR, 999077, China*

*[4]Materials and Process Simulation Center (MSC), MC 139-74, California Institute of Technology, Pasadena CA, 91125, USA*

*†These authors contributed equally to this work.*

**Corresponding Authors, email: ghc@everest.hku.hk, and wag@caltech.edu*

*ORCID: XYZ: 0009-0002-8311-0768; MT: 0000-0001-9118-5474; WJZ: 0000-0002-4328-3704; WAG: 0000-0003-0097-5716; GHC: 0000-0001-5015-0902*

**Note S1. Elementary steps in NO3RR**

The observed processes in this work during the search of minimum energy reaction pathways of $NO_3RR$ (Fig. 3c in main text) are presented here:

Proton 0: $* + NO_3^- \rightarrow *NO_3 + e^-$ (S1)

Proton 1: $*NO_3 + (H^+ + e^-) \rightarrow *NO_2 + *OH$ (S2)

Proton 2: $*NO_2 + *OH + (H^+ + e^-) \rightarrow *NO + 2*OH$ (S3)

Proton 2: $*NO_2 + *OH + (H^+ + e^-) \rightarrow *NO + *O + H_2O$ (S4)

Proton 2: $*NO_2 + *OH + (H^+ + e^-) \rightarrow *NO_2 + H_2O$ (S5)

Proton 3: $*NO + 2*OH + (H^+ + e^-) \rightarrow *NO + *OH + H_2O$ (S6)

Proton 3: $*NO + *O + (H^+ + e^-) \rightarrow *NO + *OH$ (S7)

Proton 3: $*NO_2 + (H^+ + e^-) \rightarrow *NO + *OH$ (S8)

Proton 4: $*NO + *OH + (H^+ + e^-) \rightarrow *NO + H_2O$ (S9)

Proton 5: $*NO + (H^+ + e^-) \rightarrow *N + *OH$ (S10)

Proton 6: $*N + *OH + (H^+ + e^-) \rightarrow *N + H_2O$ (S11)

Proton 6: $*N + *OH + (H^+ + e^-) \rightarrow *NH + *OH$ (S12)

Proton 7: $*N + (H^+ + e^-) \rightarrow *NH$ (S13)

Proton 7: $*NH + *OH + (H^+ + e^-) \rightarrow *NH_2 + *OH$ (S14)

Proton 8: $*NH + (H^+ + e^-) \rightarrow *NH_2$ (S15)

Proton 8: $*NH_2 + *OH + (H^+ + e^-) \rightarrow *NH_3 + *OH$ (S16)

Proton 9: $*NH_2 + (H^+ + e^-) \rightarrow *NH_3$ (S17)

Proton 9: $*NH_2 + *OH + (H^+ + e^-) \rightarrow *NH_3$ (S18)

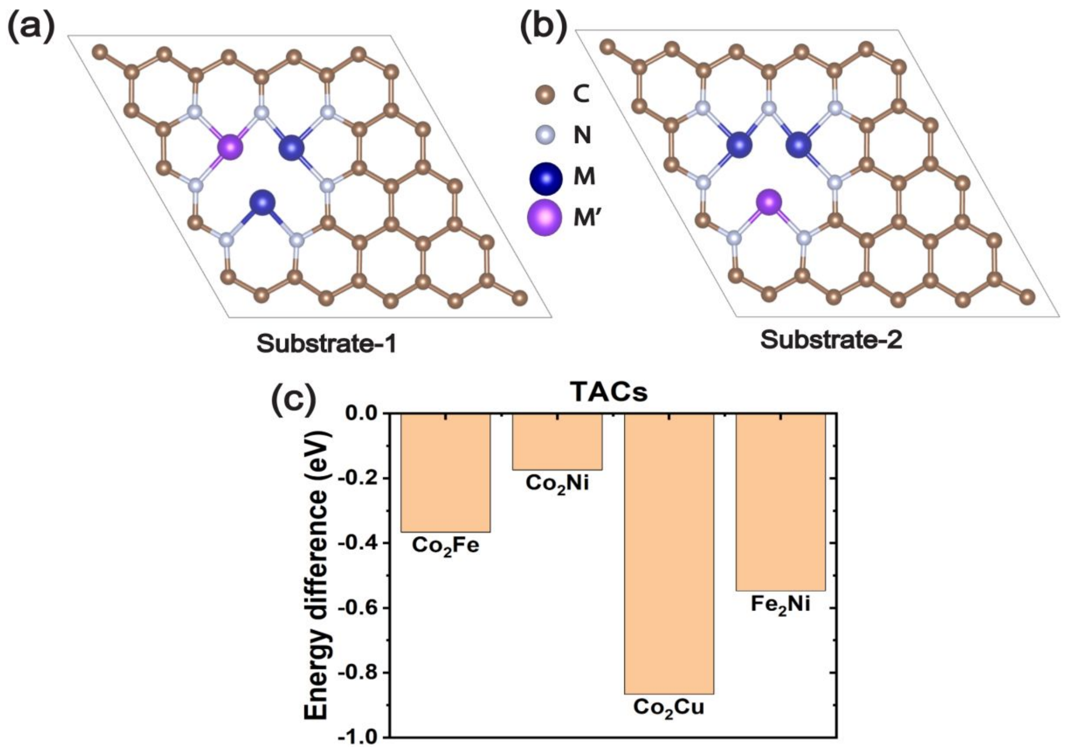


**Figure S1.** Schematics of (a-b) two kinds of substrates for bimetallic $M_2M'$-TACs and (c) the energy difference between the two kinds. The energy difference is defined as *E*(substrate-2) - *E*(substrate-1). Substrate-2 (the secondary metal atom M′ at M#3) is energetically preferable for all studied bimetallic TACs.

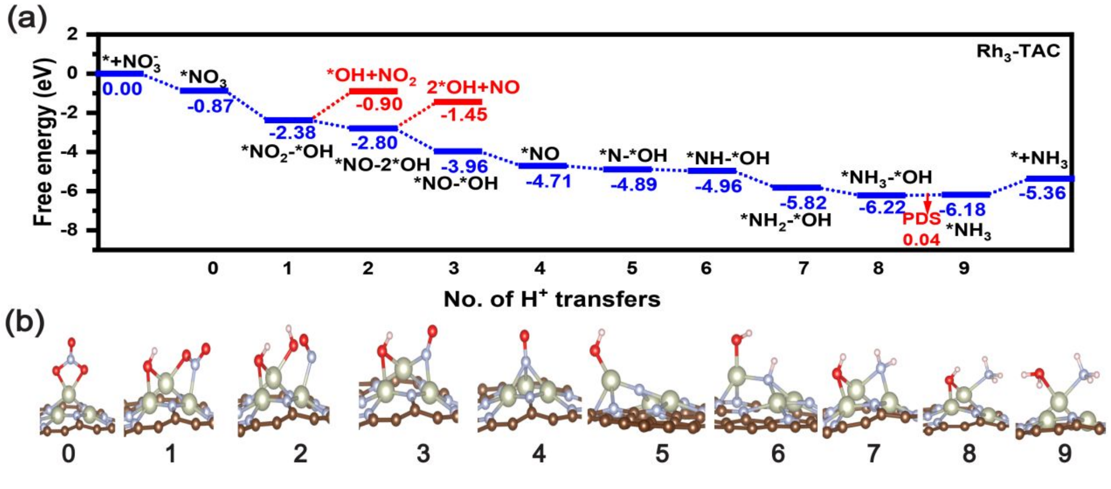


**Figure S2.** The reaction pathway (a) and the optimized configurations of intermediates (b) of $NO_3RR$ for $Rh_3$-TAC.

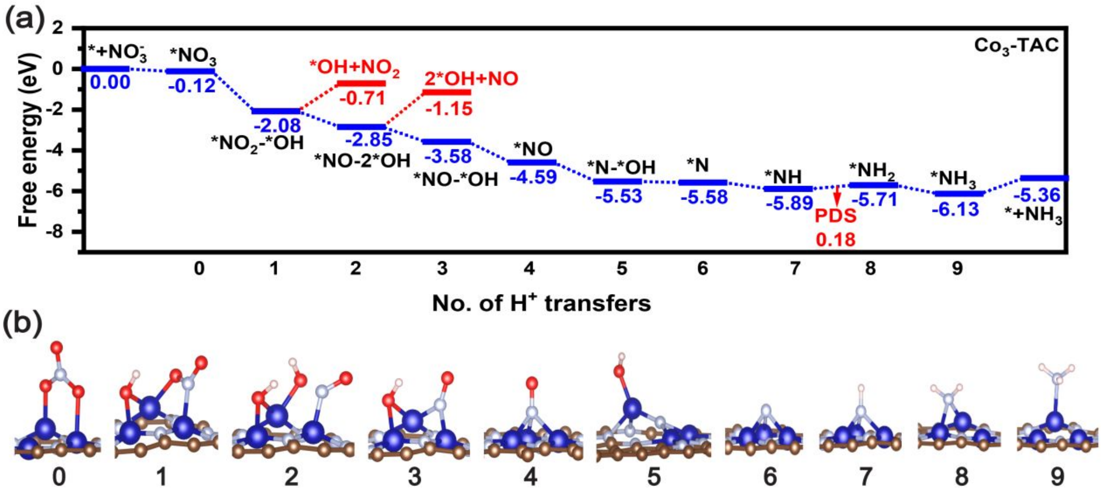


**Figure S3.** The reaction pathway (a) and the optimized configurations of intermediates (b) of $NO_3RR$ for $Co_3$-TAC without considering the influence of multiple nitrate adsorptions and pre-adsorption of $H_2O$ in the working environment.

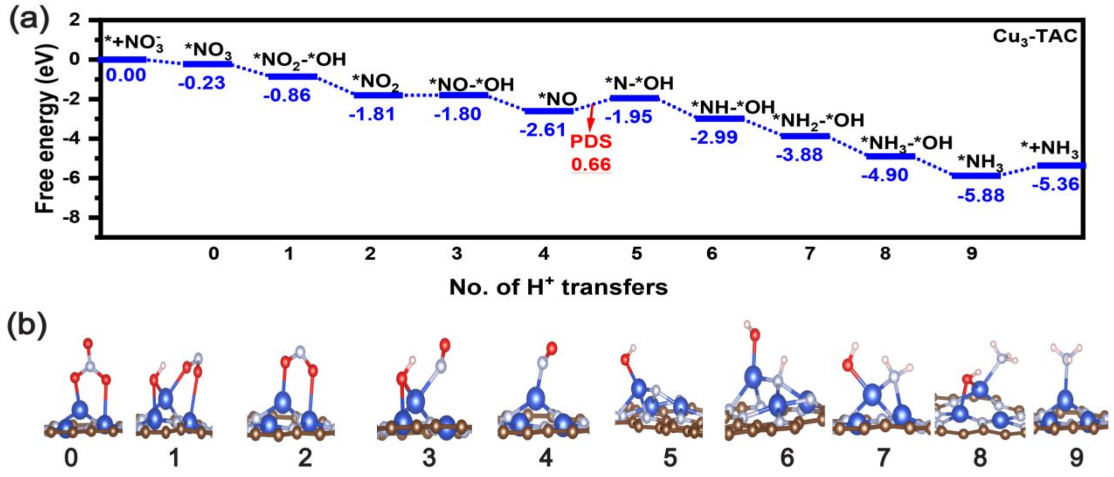


**Figure S4.** The reaction pathway (a) and the optimized configurations of intermediates (b) of $NO_3RR$ for $Cu_3$-TAC.

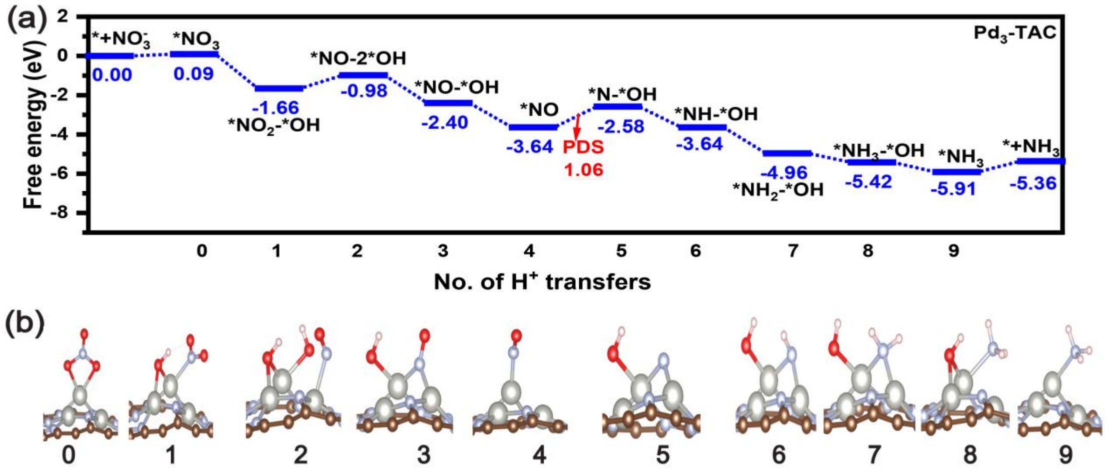


**Figure S5.** The reaction pathway (a) and the optimized configurations of intermediates (b) of $NO_3RR$ for $Pd_3$-TAC.

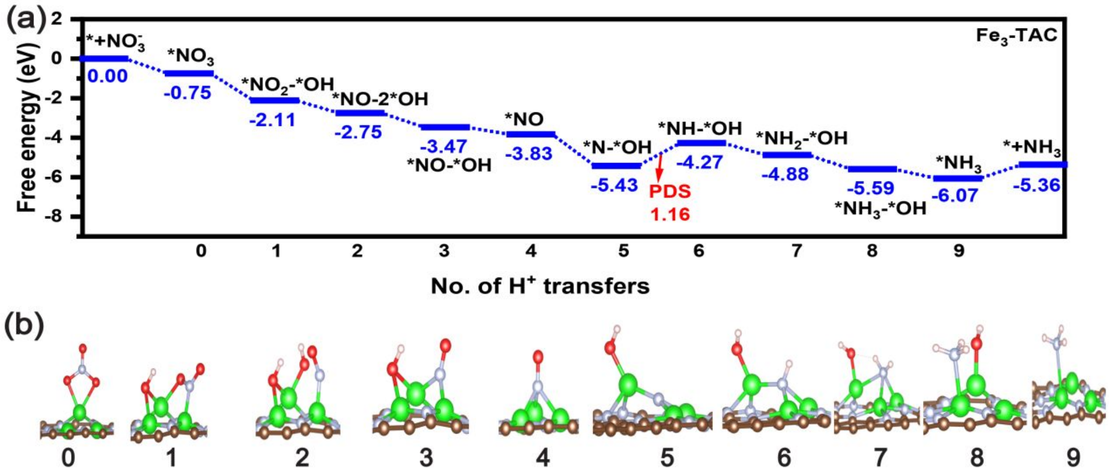


**Figure S6.** The reaction pathway (a) and the optimized configurations of intermediates (b) of $NO_3RR$ for $Fe_3$-TAC.

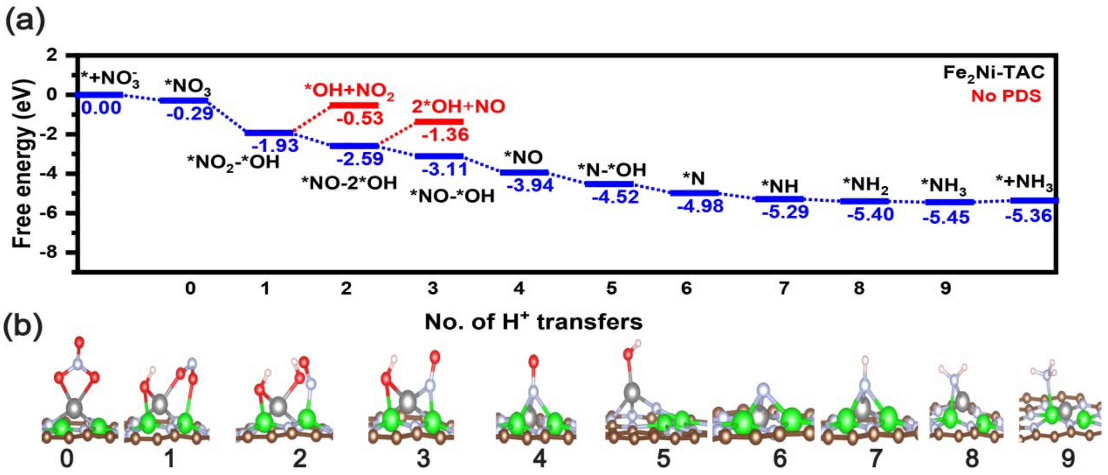


**Figure S7.** The reaction pathway (a) and the optimized configurations of intermediates (b) of $NO_3$RR for $Fe_2$Ni-TAC.

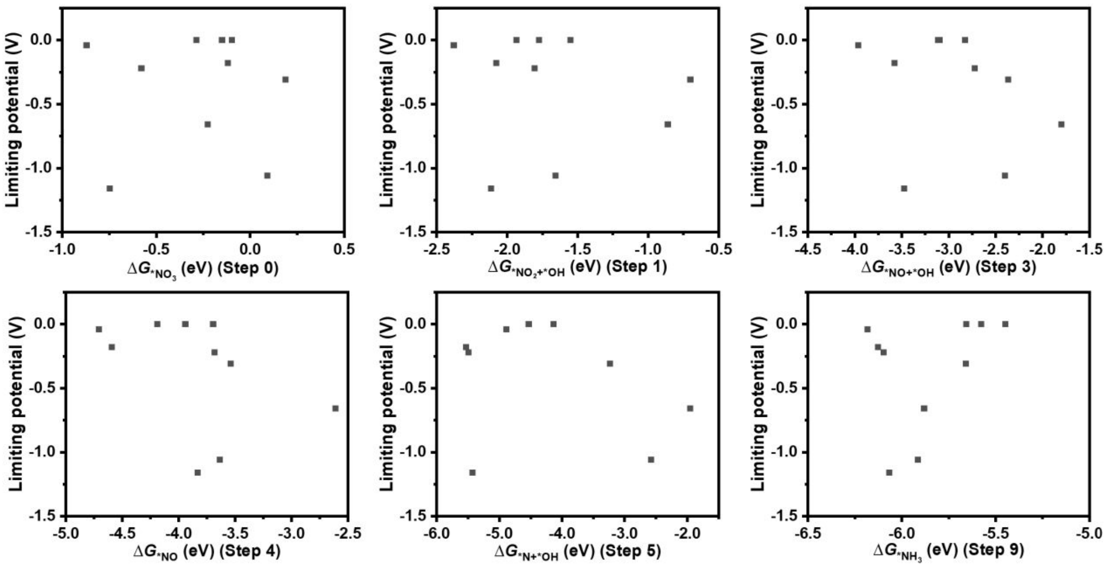


**Figure S8.** The adsorption free energies of intermediates in six protonation steps (step 0, step 1, step 3, step 4, step 5, and step 9) against limiting potential.

**Table S1.** Computed formation energies ($E_{form}$) and dissolution potential ($U_{diss}$) of homonuclear $M_3$-TACs. $E_M$ is the atomic energy of metal M in its bulk phase. $U^o_{diss\_M}$ is the standard dissolution potential of the metal M.[1] $n$ is the number of electrons transferred during the dissolution.[2] The energy of the nitrogen-doped graphene substrate $E_{NG}$ is -395.72 eV. The unit of all energies is eV, and the unit of all potential is V.

| TACs | $E_M$ | $E_{form}$ | $U^o_{diss\_M}$ | $n$ | $U_{diss}$ |
|---|---|---|---|---|---|
| $Sc_3$ | -6.25 | -3.58 | -2.08 | 3 | -0.89 |
| $Ti_3$ | -7.84 | -2.58 | -1.63 | 2 | -0.34 |
| $V_3$ | -8.99 | -1.23 | -1.18 | 2 | -0.57 |
| $Cr_3$ | -9.51 | -0.96 | -0.91 | 2 | -0.43 |
| $Mn_3$ | -9.01 | -1.30 | -1.19 | 2 | -0.54 |
| $Fe_3$ | -8.24 | -1.12 | -0.45 | 2 | 0.11 |
| $Co_3$ | -7.02 | -1.03 | -0.28 | 2 | 0.23 |
| $Ni_3$ | -5.47 | -1.39 | -0.26 | 2 | 0.43 |
| $Cu_3$ | -3.73 | -0.99 | 0.34 | 2 | 0.83 |
| $Zn_3$ | -1.11 | -1.21 | -0.76 | 2 | -0.16 |
| $Y_3$ | -6.43 | -3.59 | -2.37 | 3 | -1.17 |
| $Zr_3$ | -8.52 | -2.35 | -1.45 | 4 | -0.86 |
| $Nb_3$ | -10.22 | -0.74 | -1.10 | 3 | -0.85 |
| $Mo_3$ | -10.93 | -0.20 | -0.20 | 3 | -0.13 |
| $Ru_3$ | -9.23 | -0.03 | 0.46 | 2 | 0.48 |
| $Rh_3$ | -7.26 | -0.39 | 0.60 | 2 | 0.79 |
| $Pd_3$ | -5.22 | -0.64 | 0.95 | 2 | 1.27 |
| $Ag_3$ | -2.72 | 0.00 | 0.80 | 1 | 0.80 |
| $Cd_3$ | -0.75 | -0.49 | -0.40 | 2 | -0.16 |
| $Hf_3$ | -9.93 | -2.00 | -1.55 | 4 | -1.05 |
| $Ta_3$ | -11.81 | -0.48 | -0.60 | 3 | -0.44 |
| $W_3$ | -12.95 | 0.34 | 0.10 | 3 | -0.01 |
| $Re_3$ | -12.43 | 0.33 | 0.30 | 3 | 0.19 |
| $Os_3$ | -11.25 | 0.45 | 0.84 | 8 | 0.78 |
| $Ir_3$ | -8.85 | -0.04 | 1.16 | 3 | 1.17 |
| $Pt_3$ | -6.10 | -0.67 | 1.18 | 2 | 1.51 |
| $Au_3$ | -3.22 | 0.06 | 1.50 | 3 | 1.48 |

**Table S2.** Computed formation energies ($E_{form}$) and dissolution potential ($U_{diss}$) of bimetallic $M_2M'$-TACs. $E_{M'}$ and $E_M$ are the atomic energies of metals M′ and M in their bulk phase, respectively. The number of electrons transferred during the dissolution ($n$) is 2 for four metals involved in studied bimetallic TACs.[2] $U^{o}_{diss\_M'}$ and $U^{o}_{diss\_M}$ are the standard dissolution potential of the metals M′ and M, respectively.[1] $U_{diss}$ is taken as the minimum of $U_{diss\_M'}$ and $U_{diss\_M}$. The energy of the nitrogen-doped graphene substrate $E_{NG}$ is -395.72 eV. The unit of all energies is eV, and the unit of all potential is V.

| TACs | $E_M$ | $E_{M'}$ | $E_{form}$ | $U^{o}_{diss\_M}$ | $U^{o}_{diss\_M'}$ | $U_{diss\_M}$ | $U_{diss\_M'}$ | $U_{diss}$ |
|---|---|---|---|---|---|---|---|---|
| $Co_2Fe$ | -7.02 | -8.24 | -1.18 | -0.28 | -0.45 | 0.31 | 0.14 | 0.14 |
| $Co_2Ni$ | -7.02 | -5.47 | -1.17 | -0.28 | -0.26 | 0.31 | 0.33 | 0.31 |
| $Co_2Cu$ | -7.02 | -3.73 | -1.22 | -0.28 | 0.34 | 0.33 | 0.95 | 0.33 |
| $Fe_2Ni$ | -8.24 | -5.47 | -1.26 | -0.45 | -0.26 | 0.18 | 0.37 | 0.18 |

**Table S3.** The most stable configuration of adsorbed $*NO_3$, and the adsorption free energy of on TACs

| TACs | Adsorption configuration | $\Delta G_{*NO3}$ (eV) |
|---|---|---|
| $Fe_3$ | (3) | -0.75 |
| $Co_3$ | (1) | -0.12 |
| $Ni_3$ | (3) | 0.19 |
| $Cu_3$ | (1) | -0.23 |
| $Rh_3$ | (3) | -0.87 |
| $Pd_3$ | (3) | 0.09 |
| $Pt_3$ | (3) | 0.66 |
| $Co_2Fe$ | (1) | -0.58 |
| $Co_2Ni$ | (1) | -0.15 |
| $Co_2Cu$ | (2) | -0.10 |
| $Fe_2Ni$ | (3) | -0.29 |

**Table S4.** The limiting potential ($U_L$) and d band center ($\varepsilon_d$) on $Fe_3$, $Ni_3$, $Cu_3$-TACs.

| TACs | $U_L$ (V) | $\varepsilon_d$ (eV) |
|---|---|---|
| $Fe_3$ | -1.16 | -0.52 |
| $Ni_3$ | -0.31 | -1.48 |
| $Cu_3$ | -0.66 | -3.04 |

**Table S5.** $\Delta G_{*NO3}$, $\Delta G_{*OH}$, $\Delta G_{3*OH}$ and $\Delta G_{*H2O}$ and $\Delta G_{2*NO3}$ on $Co_3$, $Ni_3$, $Co_2Ni$, $Co_2Cu$, and $Fe_2Ni$-TACs. The unit of all free energies is eV.

| TACs | $\Delta G_{*NO3}$ | $\Delta G_{*OH}$ | $\Delta G_{3*OH}$ | $\Delta G_{*H2O}$ | $\Delta G_{2*NO3}$ |
|---|---|---|---|---|---|
| $Co_3$ | -0.12 | -0.07 | 1.04 | -0.37 | -0.55 |
| $Ni_3$ | 0.19 | 0.50 | 2.56 | 0.12 | 1.12 |
| $Co_2Ni$ | -0.15 | 0.73 | 1.45 | 0.05 | 0.02 |
| $Co_2Cu$ | -0.10 | 0.38 | 1.95 | 0.02 | 1.02 |
| $Fe_2Ni$ | -0.29 | 0.61 | 1.01 | 0.02 | -0.12 |

Note: To consider the influence posed by the pre-adsorption of OH and $H_2O$ in the working environment, we calculated $\Delta G_{*OH}$, $\Delta G_{3*OH}$ and $\Delta G_{*H2O}$. By comparing with $\Delta G_{*NO3}$ (as shown in Table S5), we find that the limiting potential on $Ni_3$, $Co_2Ni$, $Co_2Cu$ and $Fe_2Ni$-TACs will not be affected by the pre-adsorption of *OH and $H_2O$, as $\Delta G_{*NO3}$ of these TACs is lower than $\Delta G_{*OH}$, $\Delta G_{*3OH}$ and $\Delta G_{*H2O}$. The limiting potential on $Co_3$-TAC can be affected by the pre-adsorption of $H_2O$ but not *OH, as $\Delta G_{*NO3}$ of $Co_3$-TAC is higher than $\Delta G_{*H2O}$ but lower than $\Delta G_{*OH}$, $\Delta G_{*3OH}$. Therefore, we have removed $Co_3$ as the best homonuclear TAC.

To consider the influence of multiple $NO_3$ adsorptions in the limiting potential of $NO_3$RR, we calculated $\Delta G_{2*NO3}$. By comparing with $\Delta G_{*NO3}$ (as shown in Table S5), we find that the limiting potential on $Ni_3$, $Co_2Ni$, $Co_2Cu$ and $Fe_2Ni$ will not be affected by the influence of multiple $NO_3$ adsorption, as $\Delta G_{*NO3}$ is on these TACs is lower than $\Delta G_{*2NO3}$. The limiting potential on $Co_3$ can be affected by the influence of multiple $NO_3$ adsorption, as $\Delta G_{*NO3}$ of $Co_3$-TAC is higher than $\Delta G_{2*NO3}$. Therefore, we have removed $Co_3$ as the best homonuclear TAC.

# References


[1] F. Rehman, S. Kwon, C.B. Musgrave, M. Tamtaji, W.A. Goddard, Z. Luo, Nano Energy 103 (2022) 107866.

[2] W.M. Haynes, CRC Handbook of Chemistry and Physics, CRC press, 2014.

1

## Note S2. CONTCAR file of $Co_2$Ni-TAC

```
Co2Ni-TAC
   1.000000000000000
   12.2799997330000004    0.0000000000000000    0.0000000000000000
   -6.1399998665000002   10.6347917271999997    0.0000000000000000
    0.0000000000000000    0.0000000000000000   20.0000000000000000
  C    N    Co   Ni
   37    7    2    1
Selective dynamics
Direct
  0.0695979032091155  0.1441517000901559  0.1013272024304750   T   T   T
  0.0636859363350436  0.3372102303407727  0.1011561445859152   T   T   T
  0.0640853631864309  0.5314220599438579  0.0997900706030581   T   T   T
  0.0663975359761148  0.7353666747317833  0.0990839784746745   T   T   T
  0.0710399970615470  0.9416000246989782  0.1000000014999998   F   F   F
  0.2751208577712432  0.1441896750117755  0.1025169005314643   T   T   T
  0.2662808050479964  0.9333931346047297  0.1013005374543249   T   T   T
  0.4675655240256041  0.1312596011806189  0.1014982402443729   T   T   T
  0.4742467307242249  0.3372774290589505  0.1024576121002191   T   T   T
  0.4613308204829495  0.9237754439893548  0.1018859145129536   T   T   T
  0.6638049142300624  0.1270022186565056  0.0998293307313982   T   T   T
  0.6681897467511568  0.3314685511183937  0.0992400455783878   T   T   T
  0.6681098486384376  0.5314037002794366  0.1003497320918432   T   T   T
  0.6628936146700357  0.9236654149009598  0.1010832566638720   T   T   T
  0.8643038219492605  0.1312560489458216  0.0996378050850042   T   T   T
  0.8640340642384856  0.3315176052499904  0.0984729870423063   T   T   T
  0.8674021416302163  0.5340641829100742  0.0980507844519042   T   T   T
  0.8696301442826273  0.7353517406817388  0.0992070930015823   T   T   T
  0.8675910190703575  0.9333370182826407  0.1002080427728338   T   T   T
  0.1396912712834423  0.0788610023921468  0.1014613034951067   T   T   T
  0.1311158236090182  0.6674385480327933  0.1000260604293738   T   T   T
  0.1339650575325360  0.8704676154000102  0.1000262262722188   T   T   T
  0.3350722939598393  0.0688223611374499  0.1019958454529034   T   T   T
  0.3272546885992330  0.8588464670585535  0.1017491367519624   T   T   T
  0.5299950131217721  0.0592931929610306  0.1010189015367181   T   T   T
  0.5365518115180322  0.2660073711830571  0.1010034980327330   T   T   T
  0.5268892132723039  0.8533535936784049  0.1024129986406300   T   T   T
  0.7298472159641080  0.0592372304688464  0.0999629896970581   T   T   T
  0.7313530019122856  0.2619812905915350  0.0987399798086716   T   T   T
  0.7346152089480534  0.4657378196365271  0.0988070172297063   T   T   T
  0.7369723899268845  0.6673864910738833  0.1001375020518211   T   T   T
```

| | | | | | |
|---|---|---|---|---|---|
| 0.7320376470605617 | 0.8587222502428592 | 0.1008532528473023 | T | T | T |
| 0.9344499707696059 | 0.0689200014256386 | 0.1000000014999998 | F | F | F |
| 0.9301312077131400 | 0.2659931902125389 | 0.0994607340623997 | T | T | T |
| 0.9319211795710476 | 0.4657886075959766 | 0.0983500671118196 | T | T | T |
| 0.9339543563008454 | 0.6672631938093021 | 0.0983673710445422 | T | T | T |
| 0.9371100068319222 | 0.8705499768576601 | 0.1000000014999998 | F | F | F |
| 0.2593167920213060 | 0.7286807938403044 | 0.1016614660543329 | T | T | T |
| 0.1307459754021471 | 0.4679507382431077 | 0.1016291113826296 | T | T | T |
| 0.4637220411864371 | 0.7269113040267359 | 0.1042522704424293 | T | T | T |
| 0.6698476514220185 | 0.7285006285901814 | 0.1013997772210097 | T | T | T |
| 0.5379615731556148 | 0.4680236015908453 | 0.1024105959120752 | T | T | T |
| 0.3461057850651921 | 0.2748198487495356 | 0.1036334301644257 | T | T | T |
| 0.1292849533852659 | 0.2747207894650876 | 0.1025206136849949 | T | T | T |
| 0.4916843584813740 | 0.5995367735299026 | 0.1041865841258060 | T | T | T |
| 0.3084259729211523 | 0.5995293061948915 | 0.1042142351099174 | T | T | T |
| 0.3003368667142213 | 0.4001538379043984 | 0.1044012639704272 | T | T | T |
